# Evaluation of Phytochemicals and Bioactive Properties in Mangrove Associate *Suaeda monoica Forssk*. ex J.F.Gmel. of Indian Sundarbans

**DOI:** 10.3389/fphar.2021.584019

**Published:** 2021-03-10

**Authors:** Madhumita Roy, Tapan K. Dutta

**Affiliations:** Department of Microbiology, Bose Institute, Kolkata, India

**Keywords:** allelochemicals, antimicrobials, antioxidants, antidiuretics, bioautography, bioactive, mosquitocidal, *Suaeda monoica*

## Abstract

Suaeda monoica Forssk. ex J.F.Gmel. (Amaranthaceae), a mangrove associate and ethno-medicinal herb of Indian Sundarbans, was investigated as a promising source of bioactive compounds. Various polar and nonpolar solvent extracts of the leaf and root-shoot parts of the plant exhibited antioxidant, antibacterial, antifungal, allelopathic, mosquitocidal, antihaemolytic and antidiuretic potential. Moreover, to meet pharmacological requirements, the antioxidant ability of the plant was validated by both chemical and biological analyses. Extraction yield and presence of different phytochemicals like phenolics, flavonoids, tannins and saponins were compared in various solvent-extracted fractions. Principle component analysis revealed that the antioxidant property present in different extracts maintained a positive correlation with the occurrence of polyphenols (phenolics, tannins and flavonoids). Biochemical evaluation, HPLC examination and GC–MS analysis showed a differential level of the presence of various phytochemicals in different solvent extracts. In contrast to mosquitocidal, antioxidant, antihaemolytic and phytotoxic properties which were observed to be dominant in polar solvent extracts, maximum antibacterial potency was detected in nonpolar *n*-hexane fractions. Overall, the plant extract is nontoxic in nature and a dose amounting to 3,000 mg/kg was well tolerated by Swiss albino mice. A combination of HPLC and GC–MS analyses showed the presence of a large number of structurally diverse phytochemicals, many of which had already been reported as insecticidal, mosquitocidal, antibacterial, herbicidal, antidiuretic, antioxidant and anti-haemolytic compounds. All these findings support that the least explored traditional edible medicinal mangrove associate S.monoica is enriched with multiple bioactive molecules and may be considered as one of the richest sources of various lead molecules of pharmaceutical importance.

## Introduction

Indian Sundarbans is a part of the world’s largest mangrove forest (Sarker et al., 2016). The unfavorable and stressful saline conditions, tidal fluctuations, herbivore pressure, hypoxia and other stressful conditions. bring about an excessive generation of reactive oxygen species (ROS) in coastal halophytes. Nevertheless, the halophytes are inherently equipped with a powerful antioxidant and metabolic system that prevents ROS-mediated cellular damage, metabolic disorders, and senescence processes by producing various types of phenolics, flavonoids and other phytochemicals (Thatoi et al., 2014). Because of the production of varied types of bioactive compounds, use of mangroves as potent source of antimicrobials (Beula et al., 2012), insecticides (Suresh et al., 2017), antidiabetic (Nabeel et al., 2010), anticancer (Eswaraiaha et al., 2019), and antioxidants (Banerjee et al., 2008; Wangensteen et al., 2009) have been reported. Among the global mangroves, *Suaeda* species of Chenopodiaceae family occupies a special position and are known to be the producers of antibacterial (Patra et al., 2009; Muthazhagan et al., 2014; Nayak et al., 2018), antifungal (Boughalleb et al., 2009), mosquitocidal (Suresh et al., 2017), anticancer (Oueslati et al., 2012), hepatoprotective (Ravikumar et al., 2011), phytotoxic (Ali et al., 2014; Khan et al., 2018), diuretic (Kakrani and Saluja, 2001) and antioxidant (Oueslati et al., 2012) compounds.

In folklore medicine, the Suaeda group of plants are used as fodder (Ishnava et al., 2011) against rheumatism, paralysis, asthma, snake-bites, skin disease and ulcer (Kathiresan and Ramanathan, 1997; Muthazhagan et al., 2014). Again, leaves of such plants are used as antiviral, antibacterial and wound healing activities (Manivachagam et al., 2008; Ravikumar et al., 2010, 2011), besides as insecticide (Fahmy et al., 2003) and antiplasmodial against *Plasmodium falciparum* (Sundaram et al., 2011). The different species belonging to the genus *Suaeda*, viz. *Suaeda maritima* (L) Dumort, *Suaeda monoica* Forssk. ex J.F.Gmel., *Suaeda pruinosa* Lange, *Suaeda* *maritima* subsp. salsa (L.) Soó, and, *Suaeda vermiculata* Forssk. ex J.F.Gmel although widely available in Indian coastal regions but have seldom been studied*.*

The *Suaeda monoica* Forssk. ex J.F.Gmel. (Family: Amaranthaceae Juss.) is endemic to India and falls under the genus *Suaeda Forssk*. Ex J.F.Gmel which is native to Europe, Asia-Tropical, Africa, America, Australasia and other countries (Hassler, 2019). The *Suaeda monoica* species is found as mangrove associate herb in the coastal zones of India and is also known as *Suaeda monoica* (syn. *Suaeda nudiflora* (Willd.) Moq) in MPNS Portal. Except a very limited preliminary phytomedicinal studies, no in-depth work has been done on this wonderful ethnomedicinal herb species of Indian subcontinent (Jena and Das, 2006). Vanga and Peddinti (2014) were the first to report antioxidant, antibacterial and anthelmintic activities of different solvent extracts of *Suaeda monoica* while Eswaraiaha et al. (2019) showed that the leaf extract of *S. monoica* possessed anticancer property and exhibited reduced proliferation of MCF 7 and HeLa cells. Anti-cancer activity in *Suaeda monoica* Forssk. ex. J.F. Gmel. was also reported with the identification of a couple of active compounds (Al-Said et al., 2017; Siddiqui et al., 2020). In India, since local people use this halophytic succulent herb as fodder for wound healing, burn the leaves to repel mosquitoes, use as antidiuretic compound and drink the juice of the leaves in diarrhea as electrolytes, a proper validation of a variety of ethnomedicinal properties of this plant is fairly warranted. In addition, the monotypic distribution of the plant has not been addressed so far, which may be due to allelopathic/phytotoxic activity of the plant, and deserves to be investigated.

With the above background, the present study has undertaken comprehensive approaches to explore and compare a wide range of bioactivities including antimicrobial, antifungal, antioxidant, antidiuretic, allelopathic, antihaemolytic and mosquitocidal properties in various solvent extracts of the leaf and root-shoot (RS) of the plant *Suaeda*. The solvent extracts exhibiting various bioactivities were investigated further to identify the presence of various principal phytochemicals using a combination of analyses including high-performance liquid chromatography (HPLC) and gas chromatography–mass spectrometry (GC–MS).

## Materials and Methods

### Chemicals and Reagents

All chemicals used in the present study were of analytical, HPLC and/or molecular biology grade and were purchased from Himedia, India, Merck, India and Sigma-Aldrich, United States and used without further purification. Standard compounds, such as 2,2-diphenyl-1-picrylhydrazyl (DPPH), 2,2′-azino-bis(3-ethylbenzothiazoline-6-sulfonic acid (ABTS), gallic acid, caffeic acid, syringic acid, *p*-coumaric acid, ferulic acid, rutin, myricetin, quercetin, kaempferol, ascorbic acid, and antidiuretic drug furosemide were purchased from Sigma-Aldrich, United States. Solvents were procured from Merck, India. Potassium ferricyanide, trichloroacetic acid (TCA), ferric chloride, hydrogen peroxide, phosphomolybdenum, potassium persulfate etc were obtained from SRL, India. Microbiological media were obtained from HiMedia laboratory, India.

### Collection and Identification of Plant Materials

**FIGURE 1:**
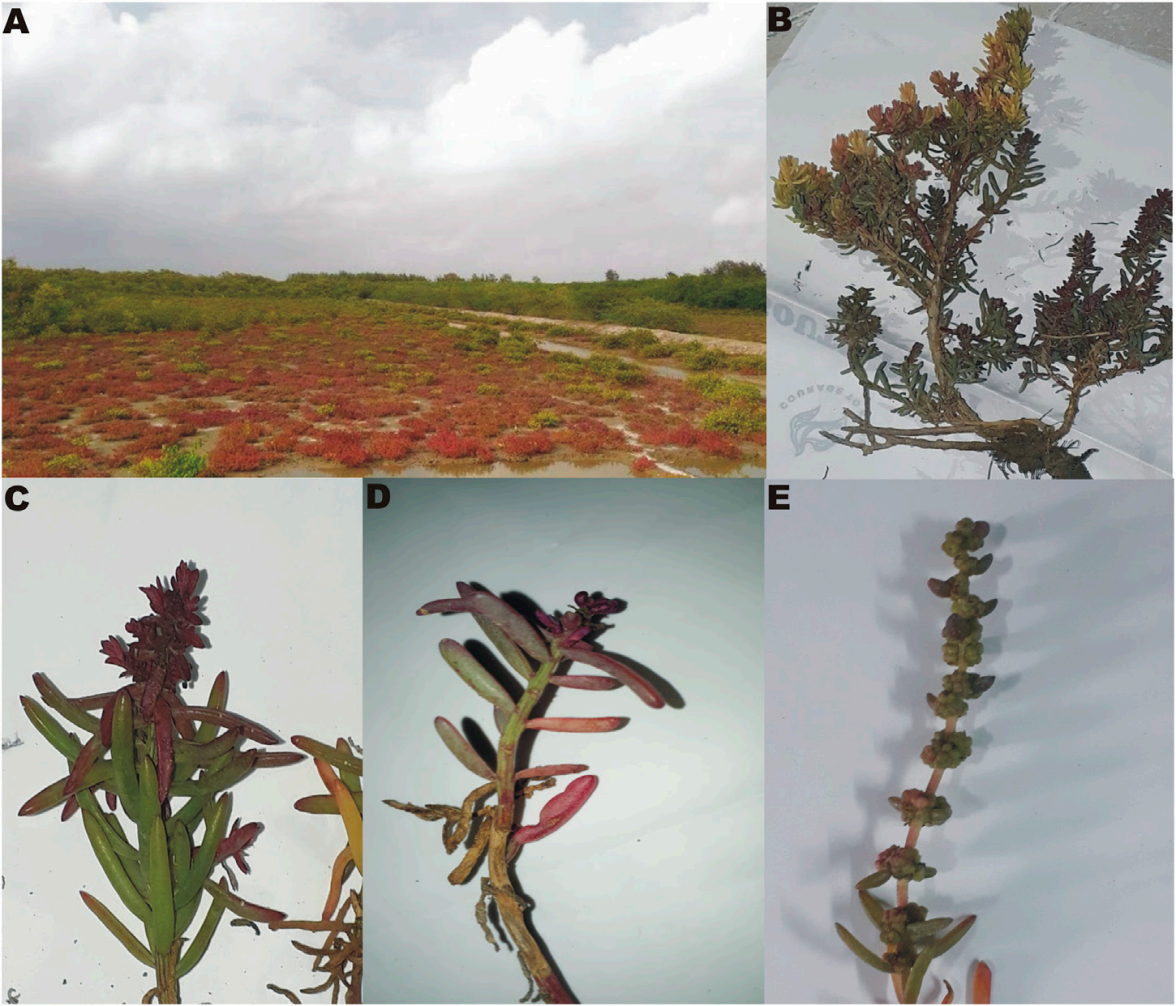
Images of *Suaeda monoica*. **(A)** Sample collection site, **(B)** isolated plant, **(C,D)** Enlarged view of leaves and stems with emerging inflorescence, **(E)** Matured inflorescence.

The plant *Suaeda monoica* was collected from Henry Island (coordinates 21°34′36.84″N, 88°17′32.28″E) of West Bengal, India where it was found as widespread scrubland with an almost monotypic species distribution (Figure 1). The plant was identified in the field by a botanist and later the herbarium sheet was deposited in the Central National Herbarium (CAL)—Botanical Survey of India [enlisted in the New York Botanical Garden (NYBG) Virtual Herbarium]. In the process, the plant was identified as *Suaeda monoica* and the associated voucher number is MR/Bose-01. The fresh leaf and RS parts of the collected plants were washed three times with sterile water under sonication and dried at room temperature. The succulent leaves were oven dried and then powdered using grinder whereas the RS parts were air dried and then grinded to powder after chopping into small pieces.

### Preparation of Solvent Extracts and Determination of Extraction Yield

The powdered plant samples (5 g each) were extracted with different solvents (250 ml each). The selected solvents with decreasing polarity index level were: water (10.2), dimethyl sulfoxide (DMSO, 7.2), methanol (5.1), acetone (5.1), ethyl acetate (4.4), chloroform (2.7), and hexane (0.1). Samples were prepared by both Soxhlet extraction (2.5 g) and maceration (2.5 g) to ensure maximum extraction of the majority of the compounds irrespective of heating. In the maceration method, the sample was dissolved in respective solvents, incubated for 4 days at room temperature with constant stirring in an orbital stirrer, and then filtered with Whatman filter paper. The filtrate solvent obtained from maceration was mixed with Soxhlet extracted solvent and evaporated to dryness in a rotary evaporator. However, for samples extracted with water or DMSO, the solvent part was lyophilized to dryness. The dried extract from original 5 g sample was then dissolved in 5 ml of methanol or suitable solvent. The extraction yield (%) was calculated as follows: Extraction yield (%) = (weight of the extract after evaporation of the solvent or freeze dried weight of the sample/dry weight of the sample) × 100.

### Qualitative and Quantitative Evaluation of Phytochemicals

Both the dried leaf and RS powder extracts, prepared in different solvents were used for the qualitative detection of the phytochemicals like phenolics, alkaloid, tannin, saponin, flavonoid, cardiac glycoside, sterol, reducing sugar, pholobatannin and steroids by established protocols (Trease and Evans, 1989; Harborne, 1998). Quantitative phytochemical test was done for the determination of phenol (Alhakmani et al., 2013), flavonoid (Chang et al., 2002), saponin and tannin (Alhakmani et al., 2013). Total phenolic content (TPC) was calculated from the calibration curve of gallic acid and expressed as mg of gallic acid equivalent (GAE)/g of dry sample. The tannin content was expressed as mg of GAE/g of dry sample. In all cases, the standard curves showed the linear regression of *R* ^2^ > 0.99. Saponin extraction and quantitative determination were carried out from 5 g of leaves and RS powder using the methods reported by Ejikeme et al. (2014). Butanol was added in the last phase of the extraction and the extracted samples were dried in a lyophilizer and the weight was measured. Each experiment was carried out in triplicates and the two-way ANOVA was used to measure statistical significance.

### Fourier Transform Infrared Analysis

Fourier Transform Infrared (FTIR) spectrum was used to identify the chemical bonds/functional groups of the components of plant extract based on the peak value ratio. 10 mg of the dried extract powder of leaf and RS samples, extracted with different solvents were encapsulated in 100 mg of KBr pellet in order to prepare translucent sample discs. The powdered sample was loaded onto FTIR spectroscope and scanned in the range of 400–4,000 cm^−1^ at a resolution of 4 cm^−1^.

### Antioxidant Activity

#### Determination of DPPH Radical Scavenging Activity

Method of Truong et al. (2019) was followed for the determination of the antioxidant activity by DPPH. The extract was serially diluted to attain concentrations of 25, 50, 100 and 150 μg/ml. One ml of each dilution was mixed with 1 ml of DPPH solution (0.004% in ethanol) and incubated at 37°C for 30 min. The absorbance of the mixture was then measured spectrophotometrically at 517 nm. Absolute ethanol was used as a negative control. DPPH scavenging activity was calculated as follows: DPPH scavenging activity (%) = [(A^0^–A)/A^0^] × 100. Where, A is the absorbance of extract and A^0^ is the absorbance of the negative control (0.004% DPPH solution). Ascorbic acid was used as a positive control. The concentration required for a 50% inhibition of DPPH (IC_50_) was then calculated by plotting the percentage of residual DPPH against the sample concentration.

#### ABTS Radical Cation Decolorization Assay

The ABTS radical cation scavenging ability was determined by following the method of Re et al. (1999) with the slight modification. ABTS radical cation was produced by incubating the mixture of 7 mM ABTS and 2.45 mM ammonium persulfate, overnight at room temperature in the dark. To determine the ABTS radical cation scavenging activity, 3 ml of ABTS radical cation solution was mixed thoroughly with 0.2 ml of different concentration (10–100 μg/ml) of plant extracts. Ascorbic acid was used as positive control. The reaction mixture was allowed to stand at room temperature for 6 min and then absorbance was measured at 745 nm. The percentage inhibition was calculated by the following formula: Percentage (%) of inhibition = [Absorbance of control–Absorbance of sample/Absorbance of control] × 100. The radical scavenging activity was expressed by IC_50_ value, calculated based on the concentration required for scavenging 50% of ABTS radical cation.

### Hydrogen Peroxide Radical Scavenging Activity

Hydrogen peroxide (H_2_O_2_) radical scavenging activities of the extracts were determined according to the method of Fernando and Soysa (2015). Briefly, water extracts of *S. monoica* (4–64 mg/ml) were added to 0.6 ml H_2_O_2_ solution (40 mM, in phosphate buffer, pH 7.4). The reaction mixtures were incubated at room temperature for 10 min and read at 230 nm against the blank solution with phosphate buffer (pH 7.4). Ascorbic acid was taken as a standard. The inhibition percentage was calculated based on the formula: Percentage (%) of inhibition = (A1 − A2)/A1 × 100. Where, A1 is the absorbance of the H_2_O_2_ and A2 is the absorbance of the reaction mixture with the extract.

### Total Reduction Capability

The total reduction ability of samples was measured according to the method of Oyaizu (1986). Ascorbic acid (3 mg, standard), was Both the human and animal studies were reviewed and approved by respective Human and Animal Ethics Committee.dissolved in 3 ml of distilled water and diluted with distilled water to prepare solutions with concentrations of 10, 25, 50, 75, and 100 μg/ml. Stock solutions of samples were prepared by dissolving 10 mg of dried extracts in 10 ml of methanol to give a concentration of 1 mg/ml and subsequently diluted to prepare solutions with sample concentrations of 10, 25, 50, 75, and 100 μg/ml. Samples (1 ml each) was mixed with 2.5 ml of 0.2 M phosphate buffer (pH 6.6) and 2.5 ml of 1% potassium ferricyanide and mixed gently. The mixture was incubated at 50°C in a water bath for 20 min. The reaction was stopped by adding 2.5 ml of 10% TCA and the mixture was centrifuged at 4,000 rpm for 10 min. From the top layer, 2.5 ml was transferred into a tube containing 2.5 ml distilled water and 0.5 ml of 0.1% ferric chloride (FeCl_3_^·6^H_2_O) and mixed thoroughly. After 5 min, the absorbance was measured at 700 nm against blank. Reducing capacity of the extracts was linearly proportional to the concentration of a sample. Phosphate buffer was used as control.

### Biological Assay of Antioxidant Activity

#### DNA Strand Protection Assay

For DNA nick test, reaction mixture was set up using 3 μl of supercoiled plasmid DNA (150 ng/μl), 10 μl of a Fenton reagent (30 mM hydrogen peroxide, 100 μM ferric chloride and 100 μM ascorbic acid) and 5 μl of plant leaf extract (0–100 μg/ml) or gallic acid (0–5 μM), volume adjusted to 20 μl by phosphate buffer (8.3 mM, pH 7.5). Reaction mixtures were incubated at 37°C for 30 min and plasmid DNA forms were separated on 0.7% agarose gels. After completion of run at 100 W, bands were visualized in Gel Doc XR system (Bio-Rad, US). Intensity of DNA bands were quantified by Quantity One^®^—Bio-Rad. Plasmid DNA incubated with Fenton reagent served as negative control while plasmid DNA incubated with the Fenton reagent and Gallic acid served as positive control. DNA protection activities of the samples were calculated from amount of supercoiled and nicked plasmid DNA using the following equations (Wang et al. 2020).

Protection of supercoiled plasmid (%) = (Intensity of supercoiled plasmid)/(Intensity of original pUC plasmid band not incubated with Fenton reagent) × 100, Protection of nicked plasmid (%) = (Intensity of nicked plasmid)/(Intensity of original pUC plasmid band not incubated with Fenton reagent) × 100.

#### Antioxidant Enzyme Assay

For antioxidant assay, tissue frozen in liquid nitrogen was crushed with a mortar and pestle. Then 0.5 g of plant material was mixed with 2 mg of PVPP (polyvinylpolypyrorolidone) and homogenized in extraction buffer (50 mM phosphate buffer, pH 7.0, containing 1 mM EDTA). The homogenate was centrifuged for 30 min at 13,000*g* and the supernatant was used for enzyme assay.

Superoxide dismutase (SOD, EC 1.15.1.1) enzyme activity was assayed by the method of Beauchamp and Fridovich (1971) based on the inhibition of the photochemical reduction of nitro blue tetrazolium (NBT). A reaction mixture containing 200 μl of 50 mM potassium phosphate buffer (pH 7.0), 300 μl of 12 mM methionine, 300 μl of 75 μM NBT, 1 ml of 50 mM Na_2_CO_3_ and 300 μl of enzyme extract was added to test mixture. In control experiment, 50 mM potassium phosphate buffer (pH 7.0) was added instead of enzyme extract. At the end, 30 μl of 1 μM riboflavin was added and the tubes were shaken and placed under a fluorescent lamp. The reaction was allowed to run for 15 min and then the tubes were immediately covered with black cloth. The absorbance was recorded at 560 nm. The non-irradiated reaction mixture kept as additional control that did not develop any color. In the test samples, due to the presence of SOD, the reaction was inhibited and the amount of inhibition was used to quantify the enzyme activity. One unit of enzyme activity corresponds to the enzyme extract volume that inhibits 50% of the photochemical reaction. The specific enzyme activity was estimated as unit per mg of protein.

Catalase (CAT, EC 1.11.1.6) activity was determined according to Aebi (1984). A reaction mixture was set up with 2,450 μl of 50 mM potassium phosphate buffer (pH 7.0), and 500 μl of 10 mM H_2_O_2_. Reaction was started with the addition of 50 μl of enzyme extract and the decrease in absorbance was recorded at 240 nm. Specific activity of the enzyme was expressed as mM H_2_O_2_ decomposed mg^−1^ protein min^−1^ by using the H_2_O_2_ extinction coefficient of 39.4 mM^−1^ cm^−1^.

Ascorbate peroxidase (APX, EC 1.11.1.11) activity was measured by the method of Nakano and Asada (1981). 50 μl of the enzyme extract was added into a reaction mixture containing 680 μl of 100 mM potassium phosphate buffer (pH 7.0), 100 μl of 10 mM EDTA and 100 μl of 10 mM ascorbic acid. Reaction was initiated with the addition of 100 μl of 100 mM H_2_O_2_. The decrease of absorbance was monitored at 290 nm and specific activity was calculated as mmol ascorbate oxidized mg^−1^ of soluble protein min^−1^ with 2.8 mM^−1^ cm^−1^ as extinction coefficient.

### Evaluation of Antimicrobial Activity and Determination of Minimum Inhibitory Concentration

Both antibacterial and antifungal assays were performed by preliminary plate-based screening and microdilution assay. For preliminary screening of antibacterial activity, agar well diffusion assay was performed (Chouhan et al., 2017) and zone of inhibition was determined. For preliminary screening of antifungal activity, the plant extract was mixed with the melted Sabouraud Dextrose agar (40°C) and then poured into petri plates. Negative control plates contained equivalent amount of solvent. When no more smell of solvent was there, small mycelial blocks were cut from the parent culture dishes and placed over the test and control plates. The plates were then incubated at 28°C for 72 h and checked for the diameter of the fungal growth in the control and test plates.

96-Well microtitre plate-based microdilution method of Eloff (1998) was used for the determination of minimum inhibitory concentration (MIC) of antibacterial and antifungal activity. For the determination of antibacterial activity, *Escherichia coli* (ATCC 25922)*, Staphylococcus aureus* (ATCC 12600), *Klebsiella pneumonia* (ATCC 700603) and carbapenem resistant *K. pneumonia* (ATCC BAA1705) obtained from the Microbial Type Culture Collection (IMTECH, Chandigarh, India) were used. Overnight grown bacterial strains were diluted with sterile Mueller–Hinton broth (MHB) to give an OD_600_ of 0.08–0.12. This was again diluted 1,000 fold to make 5 × 10^5^ CFU/ml 100 μl sterile MHB medium was added in each well of a 96-well plate. 100 μl of plant extracts (1,000 mg/ml) dissolved in methanol was added to the first well and mixed thoroughly. Then 100 µl from the first column was added in the second resulting a two-fold serial dilution and this process were continued up to 11th column (where 100 µl was discarded from the 11th column). A similar two-fold serial dilution of gentamycin (0.1 mg/ml) was used as a positive control. 100 μl of methanol was included as negative or sterility control. Finally, all wells except the wells of 12th column (negative control) received 100 µl of diluted culture. The plates were covered with parafilm and wet paper towels (to minimize evaporation) and incubated at 30°C for 24 h. Finally, 0.02% resazurin was added to indicate growth and the plates were further incubated at 30°C for 2 h. In the case of resazurin, a color gradient from violet to pink indicates increase in number of viable cells. MIC values were recorded as the concentrations in the last wells in which no color transformation in the range of blue to violet was noticed. To determine the minimum bactericidal concentration (MBC), 5 µl content from each well showing no growth were inoculated on MHB agar media aseptically and incubated at 37°C for 24 h and checked for the presence of growth. The lowest concentration of the extract that showed no bacterial growth following incubation was noted as the MBC.

Both the human and animal studies were reviewed and approved by respective Human and Animal Ethics Committee.Antifungal activity was performed against *Fusarium oxysporum*, *Alternia solani* and *Pleospora herbarum* in Sabouraud Dextrose agar. CzapekDox broth was used instead of MHB and before performing the test; fungal spore suspensions were prepared and used to achieve desired dilutions according to method described by Eloff (1998). After 48–72 h of incubation, 20 µl of 0.02% filter sterilized INT [2-(4-iodophenyl)-3-(4-nitrophenyl)-5-phenyl-2*H*-tetrazolium)] solution was added and incubated for another 24 h and observed for the formation of red colored formazan compounds in wells showing fungal growth. While colorless test wells were supposed to indicate antifungal activities of extracts, MIC values were recorded as the lowest concentrations that inhibited fungal growth after 48 h of incubation. To determine the minimum fungicidal concentrations (MFC), 50 μl of sterile Czapek-Dox broth was added to all the clear wells and further incubated at 30°C for 24 h. Then, 20 μl was plated from each well and incubated for further 72 h. The MFC was regarded as the lowest extract concentration that did not yield any fungal growth on the solid medium used.

### Thin Layer Chromatography Analysis and Bioautography

Phytochemical constituents of the plant extract were analyzed by thin layer chromatography (TLC) as described by Elisha et al. (2017) using chloroform:ethyl acetate:formic acid (8:1:1, v/v/v). Plates were visualized under UV light and Rf values of the bands were calculated by dividing the distance migrated by the bands with the distance migrated by the solvent front from the point of application. For bioautography, the developed plates were sprayed with the MHB broth-grown overnight culture of *E. coli* and incubated overnight at 37°C. After incubation, plates were sprayed with 2 mg/ml solution of INT and further incubated for 2–4 h at 37°C. Clear zones on the chromatograms indicated inhibition of growth by the separated antimicrobial compounds.

### Determination of Allelopathic Activity

Allelopathic activity was determined according to the method described by Bich and Kato-Noguchi (2014). The dried solvent extracts of plant were dissolved in methanol and applied on sterile filter paper, placed in disposable petri dishes. The methanol was evaporated under fume hood and filter paper was moistened with 0.8 ml of 0.05% (v/v) Tween 20. Ten seeds of tomato (*Solanum* lycopersicum L.) and common pigweed (*Amaranthus viridis*) were surface sterilized (dipping them in 1% sodium hypochlorite for 2 min followed by rinsing with autoclaved distilled water) and placed into the petri dishes containing test material. Controls were treated exactly as described above; with the exception that 0.2 ml methanol was used instead of *S.* extracts. Germination percentage was determined and the length of roots and shoots of the germinated seedlings were measured after 48 h of incubation in dark at 25 °C. Inhibitory activities was determined by the following formula: Inhibitory activity (%) = [(control plant length - length of plant incubated with extract)/control plant length] × 100. The dry weight and length of the seedlings were evaluated by millimeter scale. The bioassay was repeated three times using a randomized design with 10 plants and was subjected to analysis of variance (ANOVA).

### Determination of Anti-haemolytic Activity

Following approval from Institutional Human Ethics Committee, blood samples (5 ml each) were obtained from six healthy human consented volunteers, who had no report of using non-steroidal anti-inflammatory drugs for last 4 weeks. The blood cells were centrifuged at 1,000*g* for 5 min, washed with 0.2 M phosphate-buffered Saline (PBS), and reconstituted as 10% suspension in 0.5% saline (10 times volume with respect to the original). The suspension was mixed with two different concentrations (500 and 1,000 μg/ml) of the plant extracts dissolved in PBS. The reaction was incubated at 37°C for 10 min. Then 0.5 ml of 0.3% H_2_O_2_ (prepared in PBS) was added in each tube and the reaction mixtures were incubated at 37°C for 2 h. The supernatant was collected by spinning down the mixture at 1,000*g* for 10 min. The hemoglobin content of the supernatant was determined by measuring absorbance at 540 nm. One negative control was used where 500 µl extract was replaced with 500 µl of PBS. The percent of haemolysis and membrane stabilization was measured by the following formula: Haemolysis % = (Absorbance of test sample/Absorbance of control) × 100 and inhibition of haemolysis or membrane stabilization % = 100 − [(Absorbance of test sample/Absorbance of control) × 100]. Absorbance of the samples was also measured without addition of H_2_O_2_ to evaluate the direct haemolytic effect of the crude extracts. In this case, water and PBS were used as positive and negative control, respectively and extracts were dissolved in water.

### Mosquito Larvicidal Activity of *S. monoica*

The larvicidal activity of the crude extracts of *S. monoica* leaf and RS parts were tested based on the method recommended by the World Health Organization (WHO) (2005). Twenty-five fourth instar larvae of *Culex quinquefasciatus* were transferred to plastic cups containing 249 ml of water and 1.0 ml of the desired plant extract in appropriate dilutions (10–500 mg/l). An equal number of controls were set up simultaneously using tap water. Dead larvae were counted following 24 h post-treatment, and the percent mortality was reported from the average of three replicates using the formula: Percentage of mortality = Number of death larvae/Number of larvae introduced × 100. The LC50 (lethal concentration that kills 50% of the exposed larvae) and LC90 (lethal concentration that kills 90% of the exposed larvae) values were calculated based on mortality data by using probit analysis according to Abbott’s formula (Abbott, 1925). Percentage of mortality = Number of death larvae/Number of larvae introduced × 100. Statistical significance was measured by one-way ANOVA from triplicate experimental outcome.

### Screening for Diuretic Activity and Testing for Acute Oral Toxicity

The *S. monoica* leaf extracts obtained using methanol, hexane and water extracts were evaluated for their diuretic activity and acute oral toxicity (Hailu and Engidawork, 2014). Following relevant approval obtained from Institutional Animal Ethics Committee, initially, oral toxicity was tested in 18 Swiss albino mice weighing between 25 and 30 g (5–7 weeks old) using five different doses (250, 500, 1,000, 2,000 and 3,000 mg/kg) of aqueous extract. The mice were fed through stainless steel oral gavage needles and placed in standard metabolic cages. The health of test group was compared with the control group, who were fed with equivalent amount of saline water only. The animals were then observed for overt toxicities like diarrhea, weight loss, tremor, lethargy and paralysis, periodically, for every 4 h during the 24 h period and then further monitored for 14 days for any lethality. As none of the doses appeared lethal or showed any toxicity, three lower doses of 250, 500 and 1,000 mg/kg were selected for diuretic test. Here, 33 Swiss albino mice were divided into three groups. Of which, Group I and Group II had 3 mice each, representing negative control and positive control, respectively. While Group 3, the test group had 27 mice and were divided into three subgroups each having 9 mice for three different extracts (aqueous, methanol and hexane) and three different doses (250, 500 and 1,000 mg/kg). The experimental mice were denied food for 24 h. The negative control group received normal saline and positive control group received standard drug Furosemide (10 mg/kg body weight) at a rate of 2 ml/100 g of body weight. After feeding, they were administered saline water again (2 ml/100 g body weight). The cumulative urine excreted over 5 h periods from all the groups were collected in sterile containers and stored at −20°C until further analysis. Samples showing significant differences from the control set were used for flame photometric determination of sodium, potassium and chlorine ions. In addition, total urine volume, urinary electrolyte concentration of Na^+^, K^+^ and Cl^−^, diuretic action (urinary excretion of treatment groups/urinary excretion of negative control group), diuretic activity (diuretic action of test drug or plant crude extract/diuretic action of standard drug), urinary excretion [(total urinary output/total liquid administered) × 100%], saluretic activity, natriuretic activity and carbonic anhydrase inhibition were determined (Lahlou et al., 2007). The amount of Na^+^ and K^+^ was calculated as a parameter for the saluretic activity while the ratio of Na^+^/K^+^ was calculated for the natriuretic activity. To estimate the carbonic anhydrase inhibition activity, the ratio of Cl^−^/(Na^+^ + K^+^) was calculated (Vogel, 2002).

### HPLC-DAD Analysis of Phytochemicals

High performance liquid chromatography with diode array detector (HPLC-DAD) was performed to identify different classes of phenolic compounds that have characteristic UV–visible spectral properties. Dried solvent extracts (0.5 g) were dissolved in HPLC grade methanol (1 ml) and run in a HPLC system (Shimadzu Corp, Japan) along with the standards of phenolic acids (gallic acid, caffeic acid, syringic acid, *p*-coumaric acid and ferulic acid) and flavonoids (rutin, myricetin, quercetin and kaempferol) prepared at 10 mg/ml concentrations in methanol. Compounds were separated by an analytical Phenomenex C_18_ reverse-phase column (250 ×4.6 mm, particle size 5 µm), attached to a model SIL-20A autosampler and detected by a diode array model SIL-M20A detector. Solvent A (0.05% TFA in acetonitrile) and solvent B (0.05% TFA in water) were used as mobile phases. Optimized gradient elution was performed using the following program: 5–25% A (0–30 min), 25–40% A (30–40 min), 40–63% A (40–50 min) and 63–5% A (50–60 min). The flow rate was set at 1.0 ml/min, injection volume was 20 μl, and the detector wavelength was set at 254 nm (Viapiana et al., 2016). Qualitative fingerprint profile was generated for all the solvent extracts. Quantitative profile was generated for gallic acid as it is one of the most abundant compounds in all the extracts. Quantification of gallic acid was done following method of Fernandes et al. (2015).

### GC–MS Analysis

GC separation was performed by using a Thermo Scientific model with a model PolarisQ mass spectrometer at a constant flow of helium (1 ml/min). Initial oven temperature was set to 70°C and a hold for 1 min, followed by a ramp to 76°C at 1°C/min, a second ramp at 6°C/min to 350°C and a hold at 350°C for 1 min. The post run temperature was set to 325°C while the ion source was kept at 250°C and both the inlet temperature and the transfer line temperatures were kept at 340°C. Mass analyzer was used in full scan mode, scanning a range from m/z 40–800 at a scan time of 250 ms. Mass spectrometer was operated at an electron ionization energy of 70 eV. Nonpolar Durabond DB-5MS column (30 m length, 0.25 μm film thicknesses, 0.25 mm I.D, Agilent J&W) was used for the analysis of various solvent extracted samples. In addition, for methanol extracted sample, polar DB Wax column (Agilent J&W) was also used for the analysis of polar compounds. The interpretation of mass spectral data was made based on the combined Wiley Registry 11th edition/NIST 2017 Mass Spectral Library attached to the GC–MS instrument, which is one of the most comprehensive mass spectral libraries, having more than 973,000 searchable chemical structures and over 770,000 unique compounds. The criterion for peak assignment was adopted when a match quality value was higher than 80%. Moreover, the fragmentation patterns of the reported constituents were also compared with available published data.

### Statistical Analyses

The experiments were performed in technical triplicates and the data were expressed as mean ± standard deviation (SD). One or two-way (ANOVA) followed by Turkey multiple comparison was used for statistical analysis of allelopathic, mosquitocidal, antihaemolytic activities etc. All statistical analyses were done using GraphPad Prism v. 5.0 (GraphPad software Inc, USA) and *p* < 0.05, *p* < 0.01, *p* < 0.001 or *p* < 0.0001 as appropriate, were considered statistically significant. Principal component analyses (PCAs) were performed in the correlation matrix of Minitab software and IBM SPSS software.

## Results

### Determination of Extraction Yield

**FIGURE 2:**
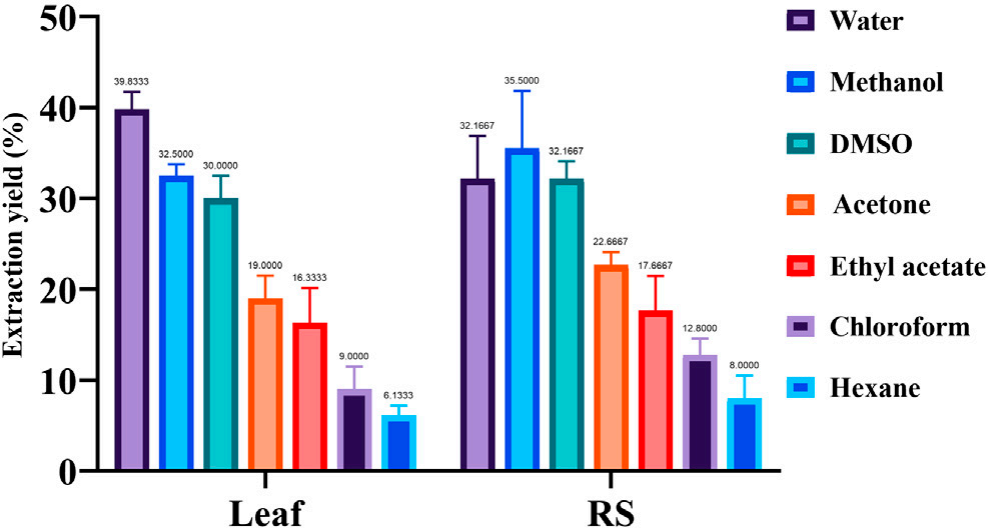
Percent extract recovery of metabolites obtained from *Suaeda* leaf and root-shoot (RS) samples using different solvents. One-way ANOVA (F 35.08; *p* < 0.0001; *R* ^2^ 0.9678) showed significant differences among means (P****) by Turkey’s multiple range test. DMSO, dimethyl sulfoxide.

Extraction yield was highest in the polar solvents and gradually decreased with a decreasing polarity of the extracting solvents. Figure 2 shows the extraction yield of the phytochemicals in the different solvents. It appears from phytochemical extraction profile that polar solvents like water and methanol can extract phytochemicals with more efficiency than that by relatively non-polar solvents.

### Qualitative Determination of Phytochemicals

**FIGURE 3:**
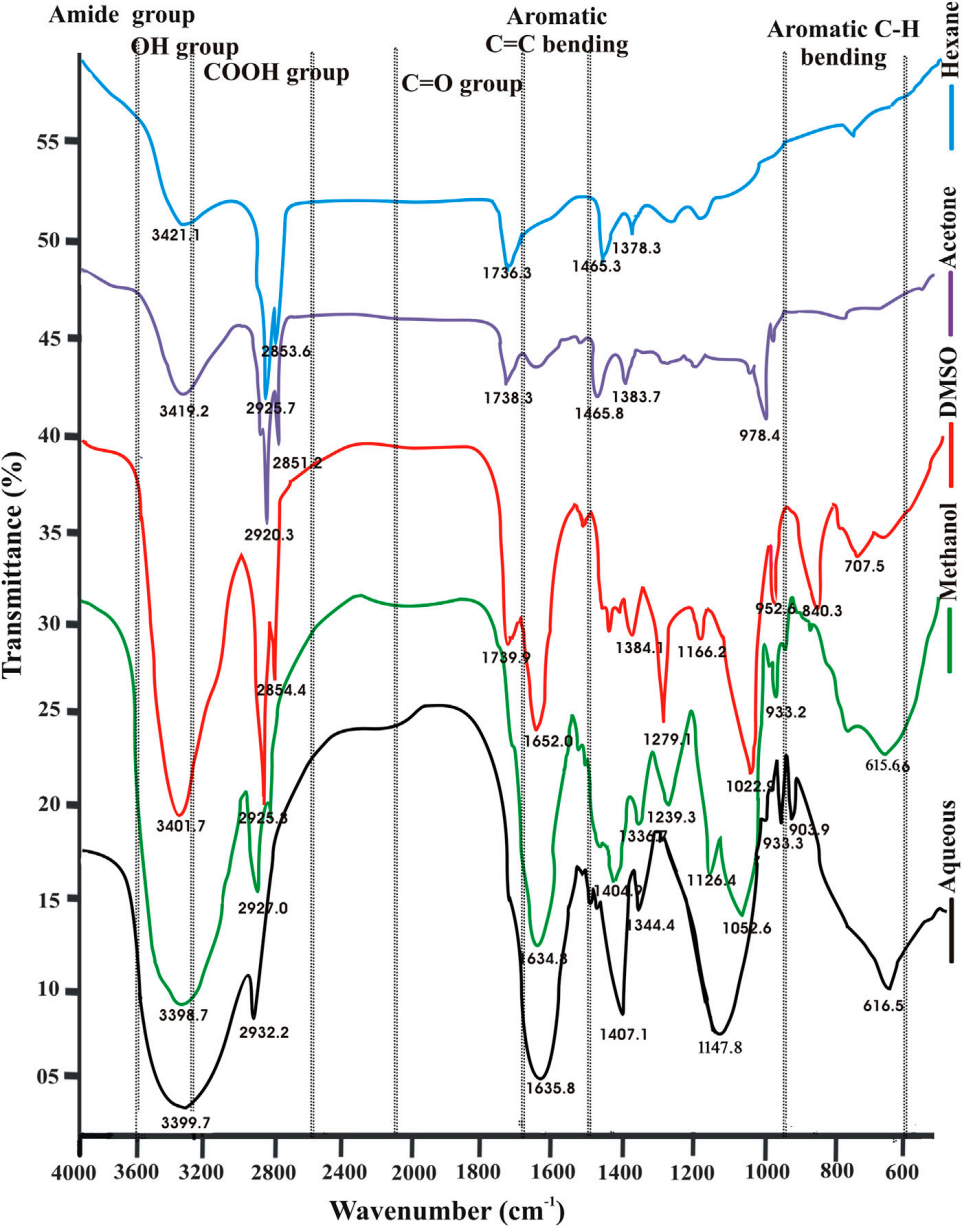
FTIR spectral profiles of various solvent extracts of leaf sample of *Suaeda monoica.*

Both the leaf and RS samples showed the presence of phenolics, flavonoids, terpenoids, tannins, saponins, steroids, phlobatannins, alkaloids, glycoside and reducing sugars in *S. monoica*. Results of biochemical tests demonstrated the presence of various phytochemicals which are shown in Supplementary Figure S1 and Supplementary Table S1. The FTIR spectroscopy results of various solvent extracts enabled to identify various chemical bonds/functional groups supporting the presence of different groups of biochemically identified phytochemicals **(** Figure 3). Presence of OH stretching (3,200–3,400 cm^−1^, strong, broad) and OH bending (1,330–1,420 cm^−1^, medium) confirmed the presence of phenolics/alcohol group. Other important groups identified by FTIR in the different solvent extracts were C-H stretching (2,840–3,000 cm^−1^, medium, sharp) for alkane, C=O stretching (1,600–1,800 cm^−1^, strong) for carbonyl group containing compounds, C=C stretching (1,600–1,670 cm^−1^) for alkene and C–O stretching (1,259–1,310 cm^−1^) for aromatic ester group. Presence of organic sulfur and alkyl halide compounds were confirmed by the presence of S=O stretching (1,290–1,320 cm^−1^) and C-halogen bond stretching (540–760 cm^−1^). Moreover, the FTIR spectra (Figure 3) showed shifting of peaks corresponding to different functional groups of various phytochemicals that were obtained with the change of polarity of the extracting solvents. Spectrum lying in the range of 3,600 and 3,200 cm^−1^ (assigned to -OH stretching of alcohol) showed very strong peaks in the polar aqueous and methanol solvents and very weak intensity peaks in nonpolar *n*-hexane. Another significant difference of FTIR spectra among different solvent systems was found in the spectral region of 1,600–1,670 cm^−1^ that indicated presence of C=C stretching of alkene groups. Spectra in this region showed sharp peaks in polar solvents and DMSO but no significant peaks in *n*-hexane. The peaks in the region of 1,720–1,740 cm^−1^ that denoted the C=O stretching of carbonyl-containing compound was prominent in nonpolar *n*-hexane but not significant in methanol and aqueous extracts. Thus, the FT-IR spectral analyses indicated a polarity-based solvent extraction at differential level of phytochemicals and that of its components containing variance in chemical bonds/functional groups.

### Quantification of Phytochemicals

**FIGURE 4:**
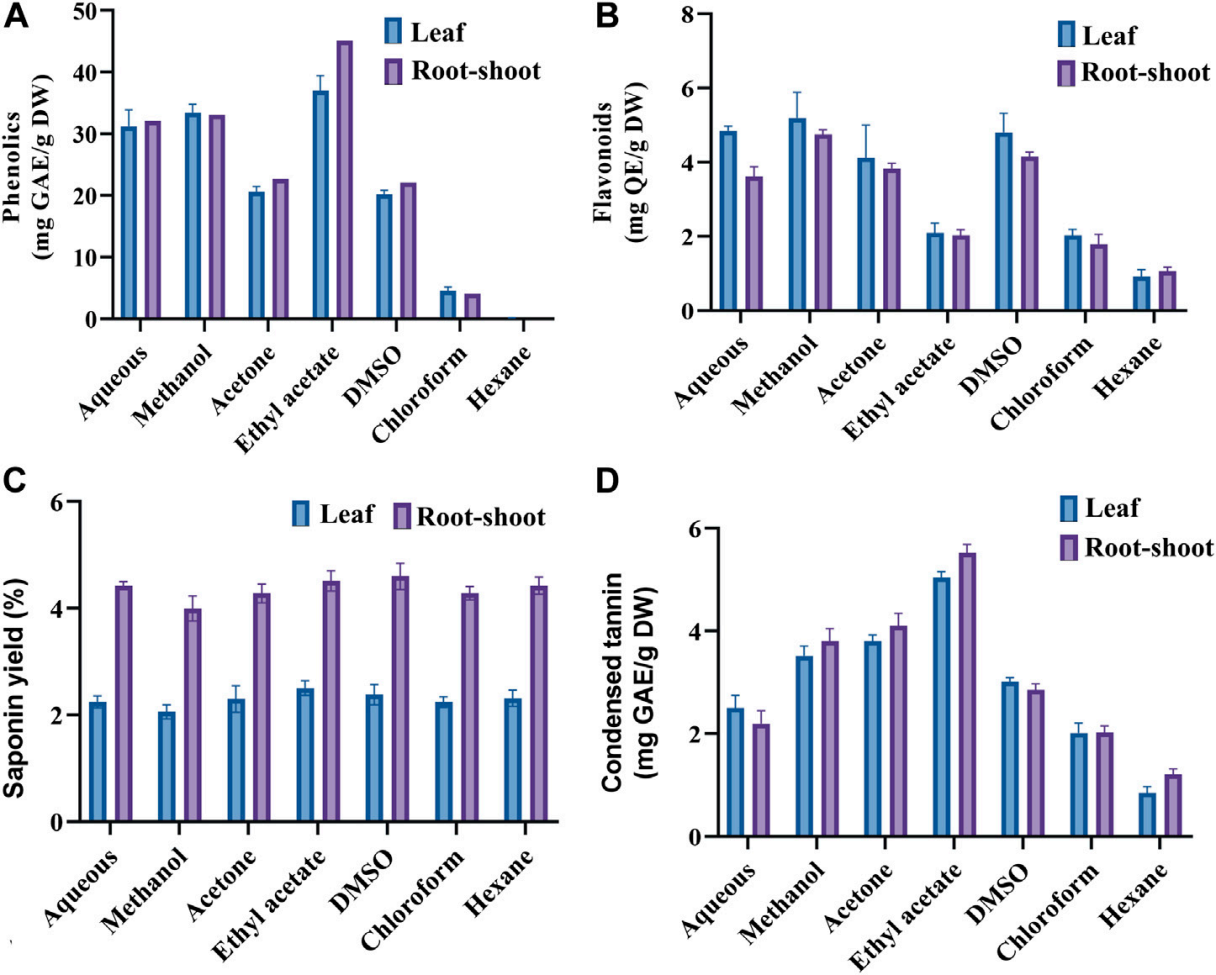
Quantification of phenolics **(A)**, flavonoids **(B)**, saponins **(C)** and condensed tannins **(D)** in the leaf and root-shoot extracts of *Suaeda monoica*. Two-way ANOVA showed significant differencesat *p* values < 0.0001 by Turkey’s multiple range test.

The effect of solvent on the total phenolics, flavonoids, condensed tannins and saponin was evaluated by ANOVA and the result is Both the human and animal studies were reviewed and approved by respective Human and Animal Ethics Committee.shown in Figure 4. It was observed that the extracts obtained using relatively polar solvent (water, DMSO, methanol and acetone) showed higher total flavonoids content (3.45–5.14 mg QE/g DW) (Figure 4B). Although, almost a similar extraction profiles were obtained for total phenolics in these polar solvents, relatively, a higher titer was obtained with ethyl acetate (37–45 mg GAE/g DW) (Figure 4A). Saponin yield was almost equivalent when extracted with different solvent systems and in all these cases, its concentration in RS sample appeared double to that present in the leaf sample (Figure 4C). Contents of condensed tannin obtained in various extracts are in the order of ethyl acetate > acetone > methanol > DMSO > water > chloroform > *n*-hexane (Figure 4D).

**FIGURE 5:**
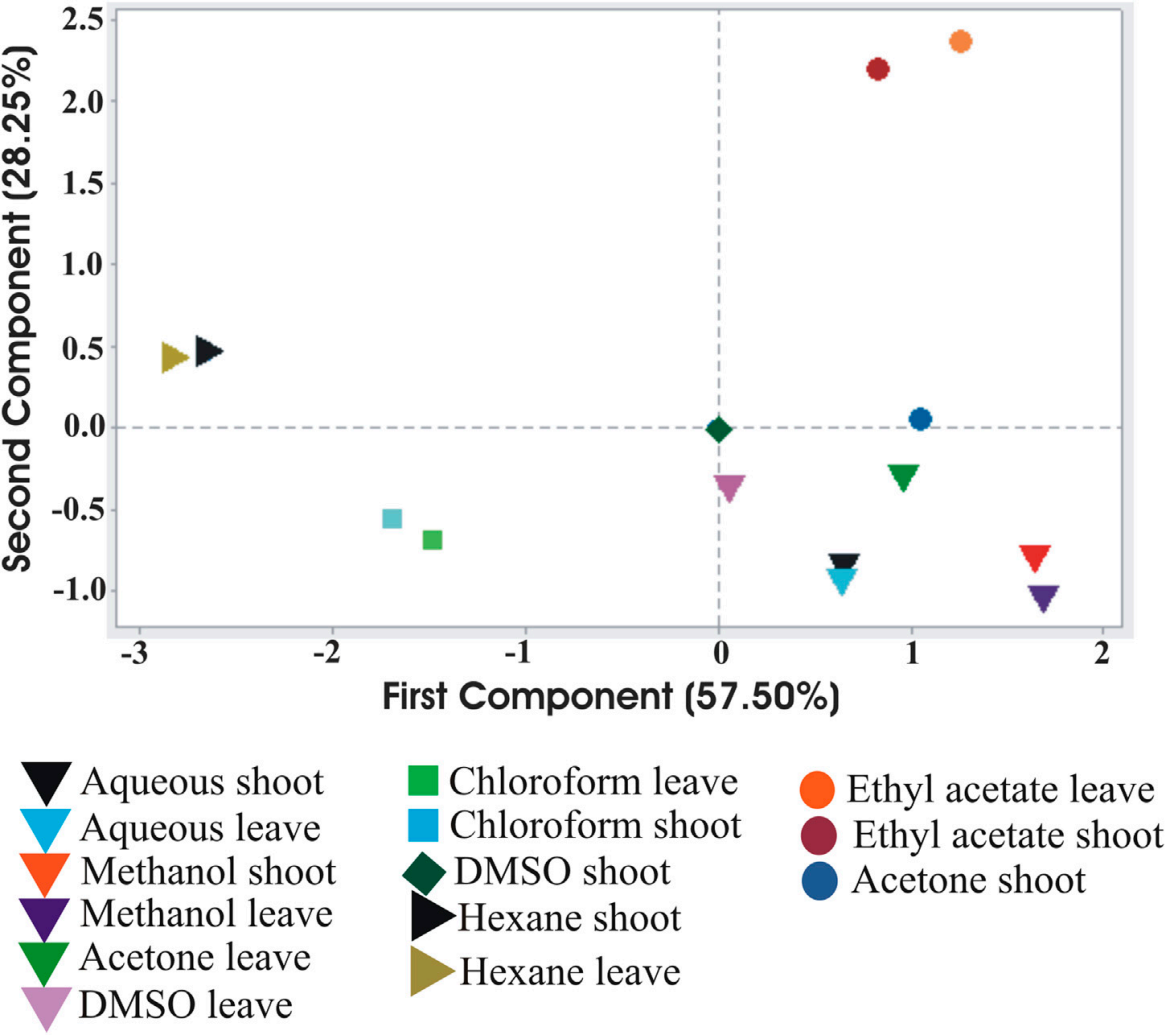
Principal Component Analysis of the interrelationship of the phytochemical components extracted from *Suaeda monoica* by different solvents. Analysis shows the first (PC1) and second (PC2) principal components.

Again, the influence of the solvents on the phytochemical extraction was evaluated based on the principle component analysis (PCA) as depicted in Figure 5. PCA performed in Minitab software confirmed variations in phytochemical components among different solvent extracts of *S. monoica* leaf and RS samples. The multivariate analysis captured 85.75% variations of the total variance on a score plot constructed with the two first PCs (PC1 57.5% and PC2 28.25%). Most of the solvent extracts of leaf and RS samples clustered in one of the quadrants of the scatter plot except chloroform, ethyl acetate and *n*-hexane extracts. As shown in Figure 5, phytochemicals obtained from water, methanol, acetone and DMSO extracts are clustered together while *n*-hexane fraction maintained highest distance from this cluster.

### Antioxidant Activity of *S. monoica*

**FIGURE 6:**
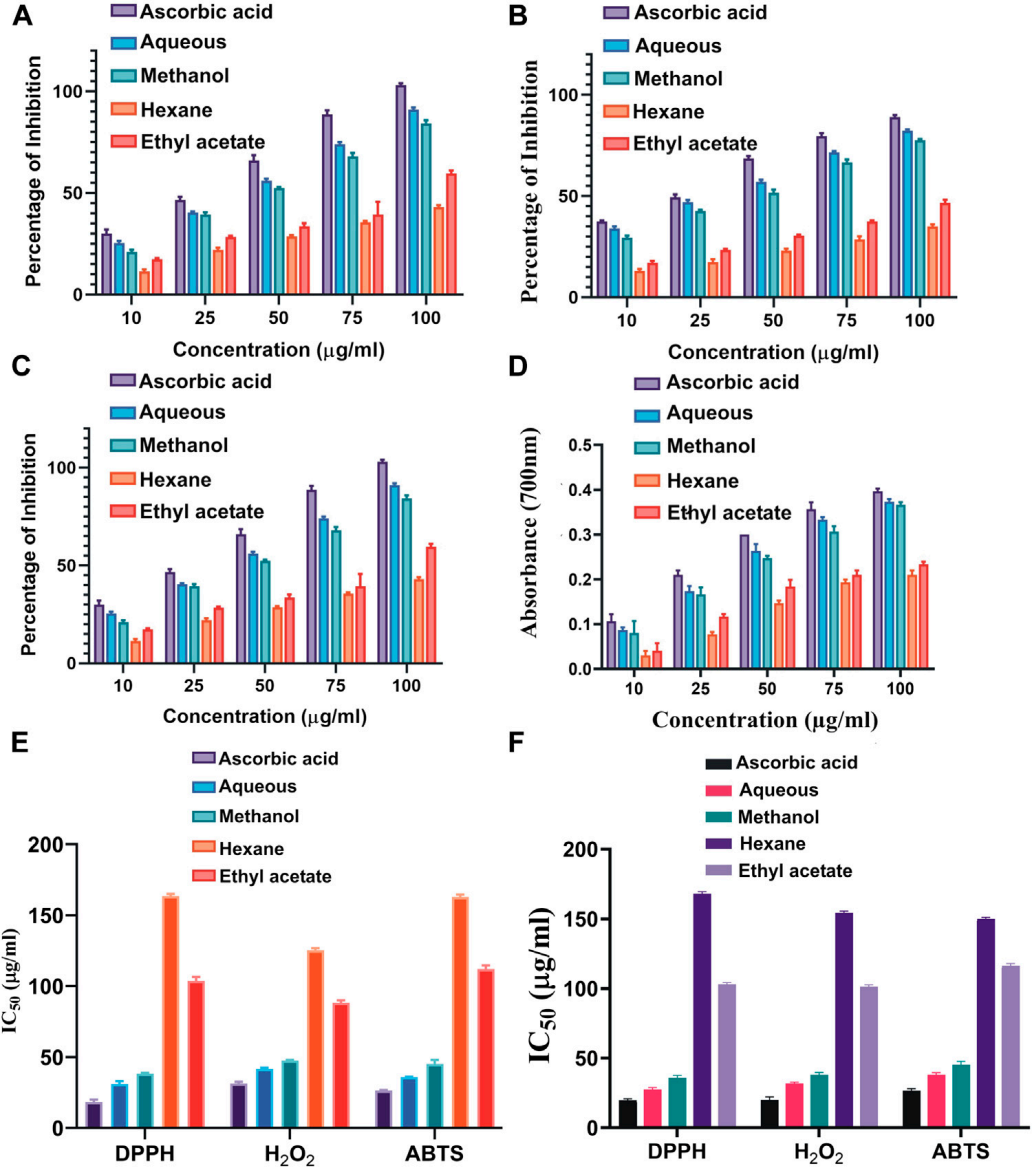
Antioxidant activity of different polar and nonpolar solvent extracts of *S. monoica* at different concentrations (10–100 μg/ml). **(A)** 2-2-Diphenyl-1-picryl-hydrazyl (DPPH) free radical scavenging activity, **(B)** ABTS radical-scavenging ability, **(C)** H_2_O_2_ radical scavenging activity, **(D)** total reductive capability **(E)** IC_50_ values of antioxidant activities of the leaves, and **(F)** IC_50_ values of antioxidant activities of the RS extracts. The bar diagrams represent the mean ± SD (*n* = 3, *p* < 0.05) for all tested dosages.

Antioxidant activities of the leaves in terms of DPPH, ABTS and H_2_O_2_ radical scavenging activities and total reduction ability are shown in Figures 6A–D, while the half maximal inhibitory concentrations (IC_50_) values of the free radical scavenging activities of the leaves and RS part are shown in Figures 6E,F. The regression graphs of both the RS and leave extracts are shown in Supplementary Figure S2. The IC_50_ values indicate that the plant polar extracts are very good radical scavengers and quite comparable with the standard ascorbic acid.

### DPPH Radical Scavenging Activity

The antioxidant activities of different solvent extracts of *S. monoica* leaves were indexed by the DPPH radical scavenging activities. Presence of an odd electron gives DPPH radical a deep purple color (Ozsoy et al., 2008). When an antioxidant compound donates an electron to DPPH, the DPPH is decolorized which is quantified by noting the change in absorbance at 515 nm. The scavenging effect on DPPH radical was varied significantly with respect to different fractions (Figure 6A). During the DPPH radical scavenging assay, the highest scavenging activity was recorded in polar aqueous and methanol followed fractions by ethyl acetate and the least in *n*-hexane fraction. This observation is in line with other studies that polar solvents are capable of extracting more antioxidant molecules than nonpolar solvents. The IC_50_ values (sample concentration that produces 50% scavenging of the DPPH radical) is inversely related to its antioxidant compounds richness (lower IC_50_ values indicate higher antioxidant activity) and is the amount of antioxidant required to decrease the DPPH concentration by 50%. Figure 6E shows the IC_50_ values of the DPPH scavenging activities of the standard ascorbic acid and the extracts. The IC_50_ values of various extracts were calculated from the regression equations of DPPH scavenging activity with ascorbic acid as standard (Supplementary Figures S2A,S2D). According to Phongpaichit et al. (2007), IC50 > 250 μg/ml is considered inactive; >100–250 μg/ml is considered weakly active; > 50–100 μg/ml is considered moderately active; 10–50 μg/ml is considered strongly active and <10 μg/ml is considered very strongly active in DPPH assay. In the present study, aqueous and methanol extracts appeared to have strong antioxidant activities and rest of the extracts have mild antioxidant activities. The plant RS extracts show almost similar profile as that of leaf extract and the IC50 values of DPPH scavenging activities are shown in Figure 6F.

### ABTS Radical Cation Decolourization Assay

This method measures the loss of color when an antioxidant is added to the blue–green chromophore ABTS·+ (2,2-azino-bis(3-ethylbenzthiazoline-6-sulfonic acid)) and reduces it to ABTS. ABTS, a protonated radical, has characteristic absorbance maxima at 734 nm, which decreases with the scavenging capacity. The results from the ABTS^+^ radical scavenging ability were found to be dose-dependent and the methanol extract proved to be an effective antioxidant (Figure 6B). The IC_50_ values of the standard and the extracts of leaves and RS part are shown in Figures 6E,F respectively. Supplementary Figures S2B,S2E shows the regression graphs of the RS and leave extracts of the ABTS radical scavenging activity**.**

### Hydrogen Peroxide Radical Scavenging Activity

Hydroxyl radicals are extremely reactive free radicals formed in our body and rapidly decomposed into oxygen and water and this may produce hydroxyl radicals (OH·) that can initiate lipid peroxidation and cause DNA damage in the body. The scavenging ability of *S. monoica* extracts and the positive control ascorbic acid are shown in Figure 6E. H_2_O_2_ radical scavenging ability of extracts of *S. monoica* and ascorbic acid at the concentration of 100 μg/ml were 88 ± 2.2 and 100 ± 2.1% respectively. The hydroxyl radical scavenging activity increased with increasing concentrations of the extracts. The IC_50_ values of H_2_O_2_ radical scavenging activities of the leaves and RS extracts are shown in Figures 6E,F, respectively. Supplementary Figures S2C,S2F shows the regression graphs of the RS and leave of the H_2_O_2_ radical scavenging activities.

### Total Reduction Capability

This method is based on the principle of increase in the absorbance of the reaction mixtures due to the presence of antioxidant molecules (Jayaprakash et al., 2001). Here, the antioxidant molecule(s) form a colored complex upon reaction with potassium ferricyanide, trichloroacetic acid and ferric chloride, which can be quantified at 700 nm. Here, the reduction capability of the tested plant extracts increased with increasing concentration as shown in Figure 6D. Ascorbic acid was used as positive control. All the extracts showed a concentration-dependent increase in the reductive ability of the test samples.

### Biological Assay of Antioxidant Activity

#### DNA Nick Test

Besides antioxidant capabilities of the extracts of *S. monoica* by chemical means like DPPH, ABTS and H_2_O_2_, DNA protection activities of the extracts were also noted. DNA nicking assay offers a sensitive *in vitro* model to determine the protective ability of antioxidants to minimize DNA damage by hydroxyl radicals generated by Fenton Reagent (Burrows and Muller, 1998). By relative electrophoretic mobility on agarose gel, supercoiled and nicked plasmid forms can be distinguished directly to asses DNA damage. As presented in Supplementary Figures S3A,B, leaf extract of *S. monoica* at 0.1 mg/ml concentration, conferred 98.72% protection of the supercoiled form and 31% protection of the nicked form. Like the extracts that protected the double strand DNA, gallic acid (standard antioxidant) can effectively protect single-stranded nicked circular form from oxidative fragmentation (Ferk et al., 2011).

#### Enzyme Assay

The plant anti-oxidative stress pathway comprises of two components, the non-enzymatic and the enzymatic components. The non-enzymatic component consists of antioxidants such as tocopherol, carotenoids, ascorbate and glutathione that are free-radical-scavenging molecules whereas the enzymatic component consists of enzymes such as superoxide dismutase (SOD), catalase (CAT) and ascorbate peroxidase (APX). All these functional elements protect the plants by scavenging the ROS generated under different types of stresses including salinity stress. SOD constitutes the first line of defense against ROS and catalyses the dismutation of the superoxide radical into H_2_O_2_ while CAT (tetrameric heme-containing enzymes) destroys the damaging H_2_O_2_ into H_2_O and O_2_. SOD, CAT, and APX activities were measured in the root, shoot and leaves of the plant. In general, it was observed that root tissues showed a higher concentration of CAT and APX activities than that of above ground tissues (Supplementary Figure S3C). On the other hand, the SOD activity was highest in leaf tissue.

### Determination of Antimicrobial Property of *S. monoica*

The different solvent extracts of leaf and RS fraction of *S. monoica* showed positive antibacterial and antifungal activities based on plate based screening (Supplementary Figure S4). MIC values of the antibacterial and antifungal activities of the solvent extracts were determined against three human pathogens and three plant pathogens respectively by microdilution assay. Both the leaf and RS samples extracted with *n*-hexane exhibited highest antibacterial activity. According to the data of MIC values, obtained for various solvent extracts, it was observed that *n*-hexane showed MIC of 3.9 mg/ml against *E. coli* and 7.82 mg/ml against *S. aureus*. Saponin exhibited modest antibacterial activity against the Gram-negative (*E. coli, K. pneumonia*) and Gram-positive (*S. aureus*) bacteria. However, all the extracts were unable to kill Carbapenem resistant *K. pneumonia* but could kill Carbapenem sensitive *K. pneumonia*. Supplementary Figure S5 shows the MIC results of antibacterial activity from 96-well plate assay. The MIC values of antifungal activity against all the three tested plant pathogenic fungi *Fusarium oxysporum* (WHK-43), *Pleospora herbarum* (WHK-137), and *Alternaria alternate* (WHK 141) were also determined. However, the antifungal activity was not as strong as that of antibacterial activity. Nevertheless, the RS fraction showed higher antifungal activity than the leaf part. The MICs, 250 mg/ml, were determined for all the extracts of RS samples while 500 mg/ml for that of the leaf samples. Table 1 shows the zone of inhibition values against the three bacterial pathogens, apart from MIC and MBC/MFC values against the selected pathogenic bacteria and fungi.

**TABLE 1:** Antimicrobial activities of leaf and combined root-shoot extract of different solvent extracts of *Suaeda monoica* against bacterial and fungal pathogens.

Solvent extract	*Escherichia coli* **(ATCC 25922)**			*Staphylococcus aureus* (ATCC 12600)			*Klebsiella pneumonia* **(ATCC 700603)**			*Fusarium oxysporum* ** (WHK-43)**	*Pleospora herbarum* (WHK-137)	***Alternaria alternate*** **(WHK 141)**
	**Zone of inhibition (mm)**	**MIC (mg/ml)**	**MBC (mg/ml)**	**Zone of inhibition (mm)**	**MIC (mg/ml)**	**MBC (µg/ml)**	**Zone of inhibition (mm)**	**MIC (mg/ml)**	**MBC(mg/ml)**	**MIC (mg/ml)**	**MIC (mg/ml)**	**MIC (mg/ml)**
CHCl_3__RS	24	15.6	31.2	22	15.6	31.2	22	15.6	31.2	nd	nd	nd
CHCl_3__L	24.5	15.6	31.2	23	15.6	31.2	23	15.6	31.2	nd	nd	nd
EtOAc_RS	24	125	125	20	125	250	20	nd	nd	125	125	125
EtOAc_L	24	15.62	31.25	20	15.62	31.25	22	nd	nd	250	250	250
Hexane_RS	22	3.906	7.812	22	7.825	15.65	23	nd	nd	125	125	125
Hexane_L	22	3.906	7.812	21.5	7.825	15.65	24	nd	nd	500	500	500
Saponin_RS	21	15.6	15.6	20.5	62.5	62.5	nd	15.6	15.6	125	125	125
Saponin_L	21	15.6	15.6	21.5	7.81	7.81	nd	15.6	15.6	250	250	250
Aquous_RS	23	500	1,500	18	500	1,500	15	nd	nd	500	500	500
Aquous_L	23	500	1,500	19	500	1,500	16	nd	nd	500	500	500
Acetone_RS	19	nd	nd	19	nd	nd	18	nd	nd	nd	nd	nd
Acetone_L	19	nd	nd	19	nd	nd	18.5	nd	nd	nd	nd	nd
MeOH_OH	16	250	500	16	500	1,000	14	nd	nd	125	125	125
MeOH_L	16.5	250	500	16.5	500	1,000	14.5	nd	nd	250	250	250
Gentamycin (500 μg/ml) for antibacterial/Cycloheximide (10 mg/ml) for antifungal	40	6.25	6.25	40	6.25	6.25	40	6.25	6.25	3.12	3.12	3.12

CHCl_3_, chloroform; EtOAc, ethyl acetate; MeOH, methanol; RS, root-shoot; L, leaf; nd, not determined

### Confirmation of Antibacterial Property by Bioautography

**FIGURE 7:**
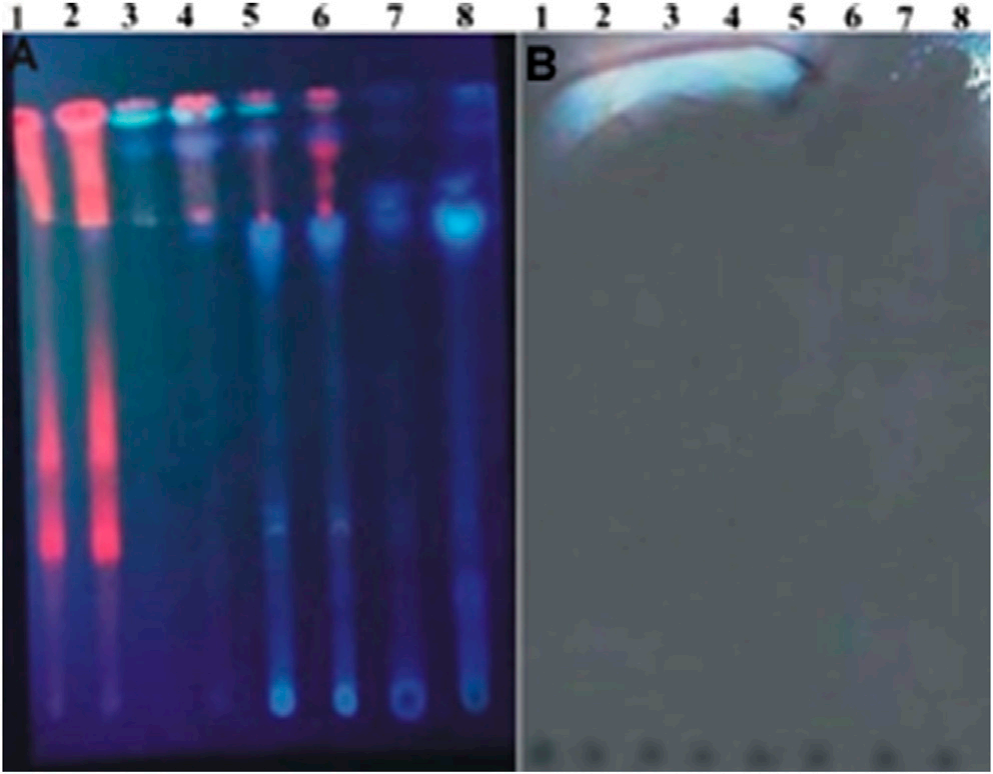
Thin-layer chromatographic outline. **(A)** Profiles of plant extract prepared with ethyl acetate (1 and 2), *n*-hexane (3 and 4), methanol (5 and 6) and water (7 and 8), **(B)** Bioautographic profile with *Escherichia coli* using identically developed chromatogram of plant extracts. White zone indicates presence of antimicrobial compounds that inhibited growth of *Escherichia coli*.

Bioautography experiment was conducted to confirm antibacterial activity present in selected extracts. The components of the extracts were first resolved by TLC. Among the different solvent systems attempted, best separation with differential banding patterns was achieved with chloroform:ethylacetate:formic acid (4:1:1, v/v/v). Figure 7A shows TLC profile of differential banding pattern of aqueous, methanol, ethyl acetate and *n*-hexane extracts of leaf and RS parts of *S. monoica*. While Figure 7B shows the bioautogram result, clearly reflecting the zone of inhibition at bands spanning at Rf values in the range of 0.90–0.92. The appearance of white areas against a purple background on the chromatograms indicates inhibition of growth of the bacterium *E. coli* due to the presence of antibacterial compound(s). It may be mentioned here that actively growing microorganisms are capable of reducing INT to a purple-black color, as appeared in the background of the TLC plate. This finding further confirms the presence of antimicrobial compounds in ethyl acetate, *n*-hexane and methanol extracts.

### Determination of Allelopathic Activity

**FIGURE 8:**
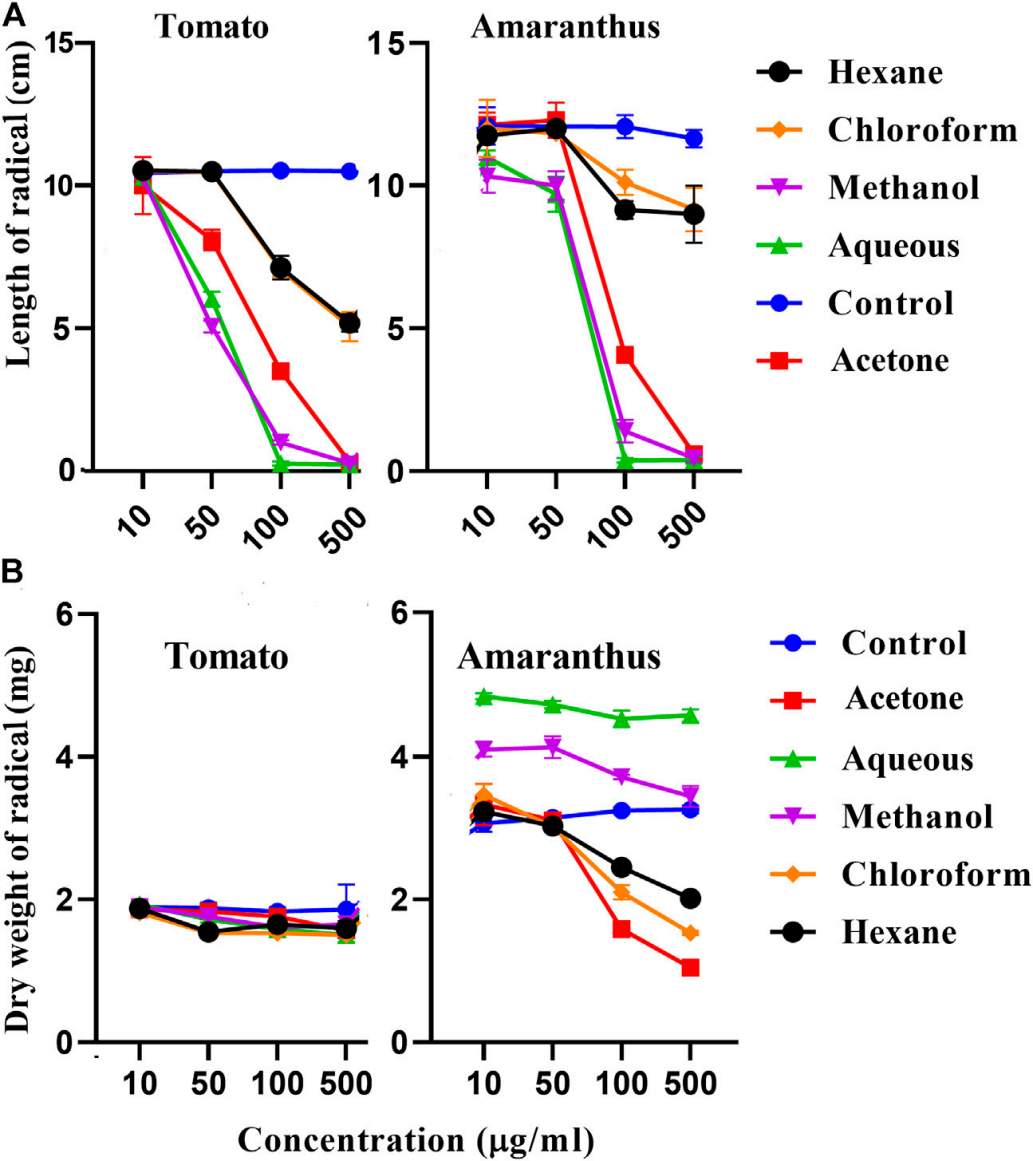
Allelopathic activity. Effect of different solvents extracts of root-shoot part of *S. monoica* on radical length **(A)** and dry weight **(B)** of germinated seedlings after 11th day of seed germination for *Solanum lycopersicum* (tomato) and *Amaranthus* (pigweed). Results were statistically significant at ***p value < 0.001.

Allelopathy is the interference that a plant exerts on other plants by the production and release of toxic compounds into the surroundings mediated by root exudates, leaf leachates and plant litter decomposition (Arroyo et al., 2018). These compounds can directly reduce germination, growth and survival of neighboring plants because of its biological effects on cell division, membrane permeability, respiration or photosynthesis, but also indirectly by modifying the activity of associated soil microorganisms and their influence on ecosystem processes Allelopathic experiments were conducted to understand the possible reason of its distribution as monotypic species, observed at the sample collection site (Figure 1). The test conducted against one representative glycophyte (*Solanum lycopersicum L. *or tomato) and a weed species (*Amaranthus viridis L.* or pigweed that can also grow in saline habitats), showed the presence of allelopathic or phytotoxic activity in the plant *S. monoica*. Germination percentages as well as lengths and dry weights of radicals were recorded from 2nd day to 13th day of incubation. In the case of negative control, i.e., germination in the presence of water only, although 100% germination was noticed for both tomato and pigweed, germination was totally inhibited (0% germination) when supplemented with methanolic (500 μg/ml) and aqueous (500 μg/ml) extracts of the RS sample of *S. monoica*. On the other hand, acetone extract (500 μg/ml) of the same sample, brought about 50% germination in the case of tomato and 80% germination in the case of pigweed while chloroform and *n*-hexane extracts (500 μg/ml) allowed 90% germination. In all the cases, lower concentration of extracts (10–20 μg/ml) had no adverse effects on germination. Radical lengths and dry weights were affected differentially with an increase in concentration above 20 μg/ml. Figure 8 illustrates the effect of solvent extracts on radical lengths and dry weights of the emerged seedling on the 11th day of germination. It may be mentioned here that leaf extracts of *S. monoica* exhibited similar type of effect like that of RS extracts (data not shown). Supplementary Figure S6 shows the plate based bioassay of seedling growth on 7th day of germination.

### Antihaemolytic Property of *S.monoica*

The various solvent extracts of the leaf and RS part of the plant *S. monoica* showed different degrees of antihaemolytic property, similar to that reported for its close relative species *S. maritima* (Rashid et al., 2017). The polyunsaturated fatty acids (PUFA) and hemoglobin which are redox-active oxygen transport molecules and potent promoters for activated oxygen species mainly target the erythrocytes, the most abundant cells of human body. However, various drugs, heavy metals, food allergens, and other factors can also cause hemolysis. The magnitude of hemolysis appeared to be much more overwhelming, when red blood cells are exposed to any toxicant like hydrogen peroxide (Woollard et al., 2009). In the present study, the antihaemolytic experiments were conducted in triplicate to assess the prevention of oxidative damages to erythrocyte membrane by *S. monoica*. When haemolysis was induced by H_2_O_2_, addition of PBS without any extract supplementation showed complete haemolysis. On the other hand, when plant extracts, dissolved in PBS, were added individually, haemolysis was reduced to different extents in a dose dependent manner, depending on the nature of solvent extracts. A maximum inhibition of hemolysis (87% RBC membrane stabilization) was exhibited by methanolic extract of leaf sample at 1000 μg/ml in comparison to other solvent extracts (Table 2). Nevertheless, RS sample was also found to possess comparable activity (data not shown). Supplementary Figure S7 shows the direct antihaemolytic effects of the solvent extracts dissolved in water (without H_2_O_2_ induced haemolysis). Here, addition of PBS as negative control did not instigate any osmotic lysis but addition of water as positive control, did affect complete osmotic lysis of the RBC making the supernatant red in color (due to released hemoglobin). But the test samples containing plant extracts dissolved in water, turned the supernatant pale yellow to green in color, clearly reflecting the presence of molecule(s) with membrane stabilization property that prevented lysis of the RBC. These experiments also indicated that with the exception of DMSO and ethyl acetate, all other solvents were found to be capableof extracting antihaemolytic components.

**TABLE 2:** Antihaemolytic property of *Suaeda monoica.*

**Solvent extract (Leaf/root-shoot, RS)**	**Optical density of H_2_O_2_ added samples at 540 nm**		**% Inhibition of haemolysis at 500 μg/ml**	**% Inhibition of haemolysis at 1,000 μg/ml**
	500 μg/ml	1,000 μg/ml		
Aqueous (leaf)	0.8545	0.606	62.596	73.47
Aqueous (RS)	0.968	0.678	57.63	70.231
Methanol (leaf)	0.5105	0.2558	77.65	86.83
Methanol (RS)	0.7308	0.3008	68.01	86.83
DMSO (leaf)	1.8676	0.8247	18.24	63.90
DMSO (RS)	2.1800	1.1567	4.57	49.37
Ethyl acetate (leaf)	1.9978	1.3001	18.25	43.01
Ethyl acetate (RS)	2.0630	1.2565	9.69	45.00
Hexane (leaf)	1.2537	0.605	45.12	73.51
Hexane (RS)	1.2467	0.606	45.427	73.47
Acetone (leaf)	1.468	0.8798	35.74	61.49
Acetone (RS)	1.9179	0.9967	16.04	56.37
PBS (negative control)	2.2845		0	

### Mosquito Larvicidal Activity of *S.monoica*

Like *Suaeda maritima* (Suresh et al., 2017), *Avicennia marina (Forssk.) Vierh.* (Balakrishnan et al., 2016) and many other mangrove plants of Indian Sundarbans (Thangam and Kathiresan, 1991), *S. monoica* plant was also found to possess significant mosquitocidal activity. In this context, plant samples are normally classified into effective (LC_50_ less than 100 mg/l), less effective (LC_50_ within 100 and 200 mg/l) and ineffective (LC_50_ more than 200 mg/l) based on their mosquitocidal activities (Suresh et al., 2017). The methanol, water, DMSO and acetone extracts of the leaf sample of *S. monoica* were found effective against the larvae of *Culex quinquefasciatus* with LC_50_ of 49.33, 68.17, 87.21, and 98.69 mg/l, respectively. Apart from above, ethyl acetate extracts showed intermediate activity with LC_50_ of 175.8 mg/l while the LC_50_ of the remaining solvent extracts were ineffective with LC_50_of more than 200 mg/l. The effectiveness of the leaf sample, extracted with different solvents and tested against the larvae of *Cx. quinguefasciatus* is presented in Supplementary Table S2.

### Determination of Acute Toxicity and Diuresis Effect

The Swiss albino mice used in the present study, showed no toxicity in the dose range of 150–3,000 mg/kg of various extracts as evidenced by the absence of tremor, weight loss, paralysis, lethargy or diarrhea. Again, none of the treated mice showed any stress symptom or got expiredwithin the observed period of 1 month, suggesting that the LD_50_ value must be greater than 3,000 mg/kg.Diuresis experiment with selected solvent extracts (aqueous, methanol and *n*-hexane) showed a positive diuresis activity in a dose dependent manner (Table 3). Diuretic activity is considered to be good, if it is more than 1.50; moderate, if it is between 1.00 and 1.50; little, if it is between 0.72-1.00 and nil, if it less than 0.72 (Hailu and Engidawork, 2014). The aqueous and methanolic extracts, administered at 1,000 mg/kg showed moderate diuretic activity while the remaining extracts displayed little or no activity under the tested dose concentrations. Concentration of electrolytes was calculated to know the type of diuresis. In the present study, along with an increase in urine volume, there was an increase in electrolyte concentration suggesting saluretic nature of remedial molecule(s) present in the plant extract but not of aquaretic type, in which case, water excretion supposed to enhance without accompanying increase in electrolytes. Also, the plant extracts showed natriuretic activity (higher Na^+^ excretion than that of K^+^). This feature is considered as an important safety profile to consider it as a diuretic agent and can serve as one of the better alternatives to synthetic diuretics like furosemide, which exerts potential adverse effects such as hypokalemiais. Moreover, the extracts showed better activity than that of standard drug furosemide in P^+^ excretion in urine. The Cl^−^/(Na^+^ + K^+^) ratio which stands for carbonic anhydrase inhibitory (CAI) activity shows a value of less than 0.8. Carbonic anhydrase inhibition activity can be excluded at ratios between 1.0 and 0.8. Carbonic anhydrase activity begins at a ratio value of less than 0.8 and descending ratio values indicate the presence of slight to strong carbonic anhydrase inhibition activity. In this study, all the extracts exhibited a CAI value of <0.8, indicating the presence of carbonic anhydrase inhibition activity. Therefore, carbonic anhydrase inhibition in diuretic action is likely to be involved in observed diuresis. The low carbonic anhydrase inhibitory activity indicated that the extracts elicited their diuretic action though a possible mechanism of increased loss of K^+^ (Vogel, 2002). This is also supported by the Na^+^/K^+^ ratios (<10) which also indicates that the extracts are not of potassium-sparing type of weak diuretic that increases urination without loss of K^+^. Results of the present study suggest that effects of some constituents in the extract on urinary electrolytes and water excretion are similar to that of furosemide. It may be mentioned here that diuretics are useful in the treatment of cardiovascular complaints like hypertension and congestive cardiac failure. In this contest, most of the patents on diuretic drugs, use a combination of diuretic sulfonamide carbonic anhydrase inhibitors with other agents for the management of cardiovascular diseases and obesity. It is believed that such combinations exert a better therapeutic activity compared to similar diuretics that do not inhibit CAs (Carta and Supuran, 2013). Thus the plant *S. monoica* supposed to serve as a suitable candidate for the treatment of hypertension associated with renal dysfunction.

**TABLE 3:** Diuretic activity of *Suaeda monoica* extract.

**Sample description (mg/kg)**	**Volume of urinary excretion (ml)**	**Diuretic action**	**Diuretic activity**	**Urinary electrolyte concentration (m mol/L)**			**Saluretic index**			**Na^+^/K^+^ (natriuretic index)**	**Cl^−^/Na^+^+K^+(carbonic anhydrase inhibition)^**
				**Na^+^ (mEq/l)**	**K^+^(mEq/l)**	**Cl^−^(mEq/l)**	**Na^+^**	**K^+^**	**Cl^−^**		
Negative control	1.02 ± 0.055	1.0		62.69	43.13	73.44				1.45	0.977
Positive control	2.15 ± 0.327	2.10		174.77	106.35	89.15	2.77	2.46	1.21	1.64	0.32
Aqueous (250)	1.25 ± 0.119	1.225	0.58	103.28	60.50	59.86	1.64	1.40	0.81	1.72	0.37
Aqueous (500)	1.80 ± 0.0673	1.76	0.84	125.31	108.40	81.50	1.99	2.51	1.10	1.15	0.35
Aqueous (1,000)	2.20 ± 0.0744	2.15	1.03	146.91	137.71	85.37	2.34	3.19	1.16	1.06	0.30
Methanol (250)	1.27 ± 0.5201	1.24	0.59	100.06	93.11	68.06	1.59	2.15	0.93	1.074	0.35
Methanol (500)	2.00 ± 0.10	1.96	0.933	134.09	116.40	96.88	2.13	2.69	1.31	1.152	0.396
Methanol (1,000)	2.25 ± 0.09	2.20	1.05	149.95	95.16	90.75	2.39	2.20	1.23	1.57	0.37
Hexane (250)	1.05 ± 0.05	1.02	0.49	63.56	55.16	113.27	1.01	1.28	1.54	1.15	0.954
Hexane (500)	1.52 ± 0.17	1.00	0.71	127.30	116.40	126.88	2.03	2.69	1.72	1.09	0.5206
Hexane (1,000)	2.05 ± 0.17	1.32	0.957	133.96	89.36	141.84	2.13	2.07	1.93	1.49	0.635

### HPLC-DAD Fingerprint Profile

**FIGURE 9:**
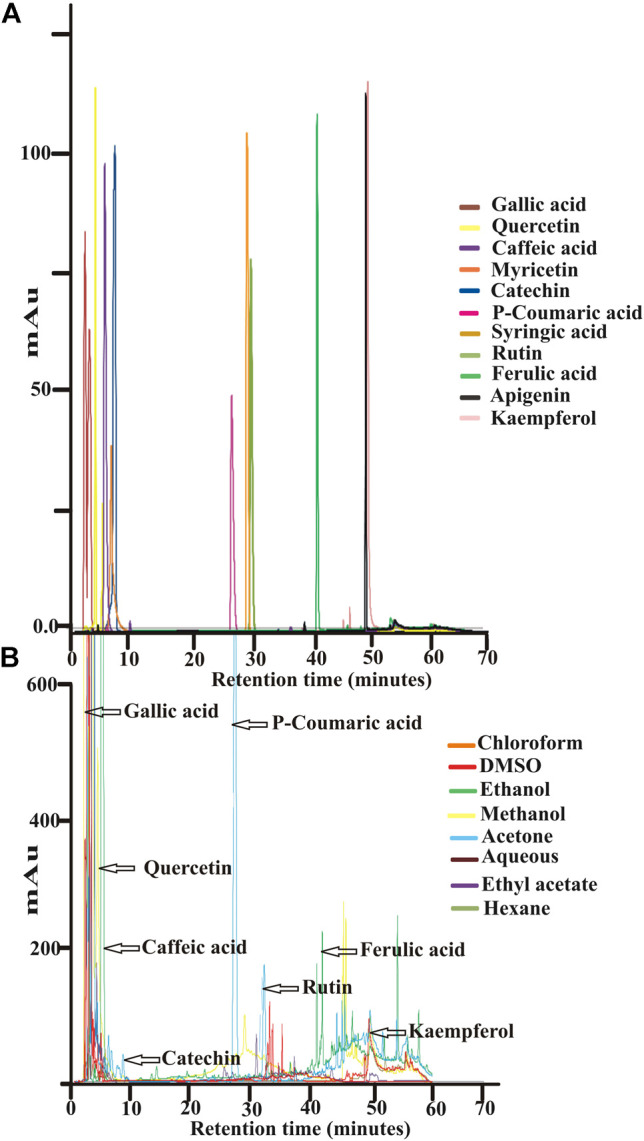
HPLC analyses. **(A)** Elution profile of various standard plant components **(B)** Merged elution profiles of various solvent extracted leaf part of *Suaeda monoica*.

HPLC-DAD analysis is one of the best methods used for chemical profiling of plant extract in a rapid and reproducible manner. In the present study, gallic acid, quercetin, catechin, caffeic acid, rutin, *p*-coumaric acid, kaempferol and ferulic acid were detected in selected solvent extracts of *S. monoica* based on the comparison of retention indexes and UV-visible spectrum of individual standards analyzed under identical conditions. Figure 9 shows the HPLC profile of various standard compounds and the merged HPLC profiles of the leaf samples, extracted with different solvents. Individual elution profiles of leaf components extracted with various solvents are depicted in Supplementary Figure S8. The RS sample gave almost similar fingerprint with little deviations in the peak areas of various components (data not shown). Based on the analysis, gallic acid was estimated to be the most abundant compound and it was found in all the solvent extracts. Kaempferol was also present in all the extracts but in smaller amounts. Other than gallic acid, some additional signature compounds were found in one or the other solvent extracts. For example, *p*-coumaric acid, quercetin and rutin were the signature compounds of medium polar solvent extract like acetone while catechin and ferulic acid were prominent in ethanol. Gallic acid was present at highest level in methanol, ethanol and aqueous extracts (3.5–4.25 μg/mg), moderate in acetone, ethyl acetate and DMSO extract (3.0–3.5 μg/mg) and lowest in chloroform and *n*-hexane extracts (1.5–2.0 μg/mg). However, it should be noted that gallic acid exists in plant in the form of free acids, esters, catechin derivatives and as hydrolysable tannins (at maximum level). Unless treated with enzymes like tannase, only the free form and the ester forms are extractable by solvent.

### GC–MS Analysis of Phytochemicals

GC–MS analyses followed by interpretation of data based on NIST/Wiley mass spectral library search revealed the identity of highest number of compounds in methanol extract followed by *n*-hexane and ethyl acetate. Among the identified compounds, a total of 90 compounds were found to have quality score higher than 80%. All these compounds were finally selected to compare their functions with similar compounds present in literature (Table 4). Based on structural configuration, the identified compounds were classified into 12 groups. The groups of phytochemicals so identified were esters (19), carboxylic acids (10), aromatic alcohols and phenols (12), alkanes (18), heterocyclic compounds (3), alkynes (2), ketones (7), alkenes (9), aldehydes (4), amines (2), ethers (2), and alkylbenzenes (2). Nevertheless, Table 4 illustrates a differential presence of compounds in various solvent extracts and also depicts the association of some of these biomolecules with functions related to antimicrobial, allelopathic and mosquitocidal properties. Analysis of methanol extracted sample using polar wax column generated the most significant peak of acetic acid which was not observed from the same sample when run using the nonpolar DB-5MS column. For the sake of elucidating a quantitative estimation, based on the GC peak area, each compound was expressed as the percentage of the total compounds, and the results of all the three solvent extracted samples are displayed in Supplementary Figure S9. The chemical structures of the major phytochemicals are presented in Supplementary Figure S10. Thus, the GC–MS analysis of various bioactive compounds from the plant extract supported our findings on the existence of a variety of bioactivities of this plant and also justifies the use of this plant for treating various ailments by traditional practitioners.

**TABLE 4:** Identification of phytochemicals from various solvent extracts of the root-shoot sample of *Suaeda monoica*.

**Serial number**	**Compound name**	**Molecular formula and molecular weight**	**CAS No**	**Retention indexes (Kovat’s Indexes) **	**Presence/absence of compounds in solvent system and peak area % in parentheses**			**Bioactivity**
					**Methanol**	**Ethyl acetate**	**n-Hexane**	
Amine class of compounds								
1	Ethanamine, 2-chloro-N,N-dimethyl-	C_4_H_10_ClN 107.582	107-99-3	5.628	0.40	–	–	Herbicide (Rodgman and Perfetti, 2016)
2	Methenamine	C_6_H_12_N_4_ 140.186	100-97-0	17.109	0.44			Bactericidal property
Ketone class of compounds								
3	2-Hexanone	C_6_H_12_O 100.16	591-78-6	5.831	–	–	0.33	Essential oil (plant volatile)–function unknown
4	3-Hexen-2-one		763-93-9	5.924	1.88	–	–	
5	2-Pentanone, 4-methoxy-4-methyl-	C_7_H_14_O_2_ 130.18	107-70-0	8.000	0.99		–	Neutraceutical (^a^)
				6.753		0.14		
6	Furyl hydroxymethyl ketone	C_6_H_6_O_3 126.11_	17,678-19-2	25.61	0.83	–	–	Ketogenic foods help to recover from cancer (Onyeaghala et al., 2015)
7	4H-Pyran-4-one, 2,3-dihydro-3,5-dihydroxy-6-methyl-	C_6_H_8_O_4_ 144.1253	28,564-83-2	14.656	1.49	–	–	A flavonoid with antioxidant and antifungal activity to inhibit growth or spore germination (Čechovská et al., 2011)
8	5,9-Undecadien-2-one, 6,10-dimethyl-, (E)-	C_13_H_22_O 194.3132	003796-70-1	24.141	-	0.20	–	A monoterpene ketone and an essential oil
9	7,9-Di-tert-butyl-1-oxaspiro (4,5)d eca-6,9-diene-2,8-dione	C_17_H_24_O_3_ 276.370	82,304-66-3	29.579	-	0.47		Diuretic, daphoretic, coronary-dilator, disinfectant and discutient (^a^)
				29.583			0.97	
Ester class of compounds								
10	Glycine, N,N-dimethyl-, methyl ester	C_5_H_11_NO_2_ 117.15	7148-06-3	6.639	0.84	–	–	Precursor to organic acid synthesis; glycine betaine is an osmoprotectant/compatible solute, i.e., a substance highly soluble in water, which protects the proteins from denaturation and enhances plant tolerance to abiotic stress (Sakamoto and Murata, 2002)
11	Trichloroacetic acid, hexadecyl ester or Hexadecyltrichloroacetate	C_18_H_33_Cl_3_O_2_ 387.812	74,339-54-1	30.548	0.98	–	–	Urinary acidifier and acidulant, inhibit production of uric acid (^a^)
				25.342			0.11	
12	Trichloroacetic acid, pentadecyl ester	C_17_H_31_Cl_3_O_2_ 373.786	74,339-53-0	28.158	0.24	–		Herbicide (Baxter et al., 1998)
				28.154			+ (0.21)	
13	Acetic acid, hydroxy-, methyl ester or methyl glycolate	C_3_H_6_O_3_ 90.0779	96-35-5	12.573	0.11	–	–	Glycolate is a part of primary plant metabolism and photosynthesis (Cui et al., 2016)
14	Carbamic acid, methyl ester	C_2_H_5_NO_2_ 75.0666	598-55-0	5.548	0.55	–	–	Insecticide, antimicrobial, herbicide (Melnikov, 1971)
15	Carbonic acid, octadecyl 2,2,2-trichloroethyl ester	C_21_H_39_Cl_3_O_3_ 445.9	1,000,314-56-3	32.184	–	3.27	–	Unknown
16	11-Octadecenoic acid, methyl ester	C_12_H_20_O_2_ 296.4879	52,380-33-3	31.530	0.18	–	–	anti-inflammatory, antiandrogenic, and anemiagenic properties (Surender et al., 2008)
17	9,12-Octadecadienoic acid (Z,Z)-, methyl ester	C_19_H_34_O_2_ 294.4721	112-63-0	31.482	9.80			Anticancer, anti-inflammatory, hypocholesterolemic, hepatoprotective, nematicide, insectifuge, antihistaminic, anti-eczemic, 5-alpha reductase (testosterone reductase) inhibitor, antiandrogenic, antiarthritic and anticoronary (^a^)
				31.469		0.70		
				31.469			1.55	
18	10,13-Octadecadienoic acid, methyl ester	C_19_H_34_O_2_ 294.47		31.469	–	–	1.55	Anti-inflammatory, hypocholesterolemic, cancer preventive, hepatoprotective, nematicide, insectifuge, antieczemic, anticancer, antiarthritic, insectifuge, antihistaminic and anticoronary (^a^)
19	9,12,15-Octadecatrienoic acid, methyl ester, (Z,Z,Z)- or methyl linolenate	C_19_H_32_O_2_ 292.5	301-00-8	31.529	5.13	–		Antiinflammatory, anti-atherogenic, hepatoprotective and reduces proliferation of tumor cells (^a^)
				31.516			1.02	
20	1,2-benzene dicarboxylic acid, mono 2-ethylhexyl ester	C_16_H_22_O_4_ 278.3435	002566-97-4	31.482	9.80			Anticancer and cytotoxic (Krishnan et al., 2014)
				31.469		0.70		
				31.469			1.55	
21	Octadecanoic acid, methyl ester or methyl stearate	C_19_H_38_O_2_ 298.5	112-61-8	31.710	3.47	–		Anti-inflammatory, antiandrogenic, cancer preventive, dermatitigenic, irritant, antileukotriene—D4, hypocholesterolemic, 5-alpha reductase inhibitor, anemiageni and insectifuge (^a^)
				31.706			1.56	
22	Benzoic acid, 4-ethoxy-, ethyl ester	C_11_H_14_O_3_ 194.2271	23,676-09-7	24.221	–	0.24	0.39	Natural Preservative against bacterial food spoilage (Arokiyaraj et al., 2018)
23	Benzoic acid, 4-hydroxy-3,5-dimethoxy-, hydrazide	C_9_H_12_N_2_O_4_ 212.2026	001443-76-1	27.960	0.38			No records found
24	Benzoic acid, 2-hydroxy-, 3-methyl butyl ester or isoamyl salicylate	C_12_H_16_O_3_ 208.2536	87-20-7	25.135	–	–	0.25	Nutrient, flavoring agent (^a^)
25	Hexadecanoic acid, methyl ester or methyl palmitate	C_17_H_34_O_2_ 270.4507	112-39-0	29.748	5.38			Antioxidant, nematicide, pesticide, lubricant, antiandrogenic, flavor, hemolytic 5-alpha reductase inhibitor, Hypocholesterolemic (^a^)
				29.74		1.53		
				29.736			2.93	
26	Nonanoic acid, 9-oxo-, methyl ester	C_10_H_18_O_3_ 186.2481	1931-63-1	25.342	–	–	0.11	Herbicide with antifungal properties (Martelloni et al., 2020) (US patent 5,683,962)
27	1,2-Benzenedicarboxylic acid, butyl cyclohexyl ester	C_18_H_24_O_4_ 304.3808	84-64-0	30.184	0.98		–	Not known
				30.184		1.73		
28	Hexanedioic acid, bis (2-ethylhexyl) ester	C_22_H_42_O_4_ 370.6	103-23-1	33.461	3.83			Major anti-algal chemical (Choe and Jung, 2002) (Choi and Jiang, 2014)
				33.478		7.64		
				33.461			9.61	
Aldehyde class of compounds								
29	Furfural	C_14_H_28_ 196.37	98-01-1	6.487	0.90	–	–	Antifungal, allelochemical (Chotsaeng et al., 2018)
30	2-Furancarboxaldehyde, 5-methyl-	C_14_H_28_O_2_ 228.376	620-02-0	9.218	0.39	–	–	Antioxidant and antiproliferative activities (Zhou et al., 2008)
31	2-Furancarboxaldehyde, 5-(hydroxymethyl) or 5-Hydroxymethyl furfural	C_6_H_6_O_3_ 126.110	000067-47-0	17.265	1.96	–	–	Antioxidant and antiproliferative activities (Zhou et al., 2008)
32	Octanal, 2-(phenylmethylene)-or Hexyl cinnamic aldehyde	C_15_H_20_O 216.3187	101-86-0	27.651	–	–	0.65	Flavoring agent, nutraceutical (has use in cosmetics) Gunia-Krzyżak et al., 2018
Aromatic alcohol and phenol								
33	Phytol	C_20_H_40_O 296.5310	150-86-7	31.964	1.52		–	Precursor for synthesis of chlorophyll, tocopherol, phylloquinol and fatty acid phytyl ester; Anticancer, antioxidant, diuretic, anti-inflammatory, antitumor, chemopreventive and antimicrobial (^a^)
				32.298		1.13		
34	Maltol	C_6_H_6_O_3 126.11_	118-71-8	25.049	0.12	–	–	Flavor enhancing nutraceutical (^a^)
35	2-Furanmethanol or Furfuryl alcohol	C_17_H_30_OSi 99.0999	98-00-0	7.041	1.44	–	–	Allelochemical (Chen et al., 2012)
36	Benzofuran, 2,3-dihydro-or Coumaran	C_8_H_8_O 120.1485	496-16-2	16.923	9.98		–	Analgesic, anticancer, anti-inflammatory and anti-diarrhoeal (Farooq et al., 2019)
				16.851		1.69		
37	Benzyl alcohol	C_6_H_5_CH_2_OH 108.137	100-51-6	11.409	1.22	–	–	Detoxicant, antiparasitic, insecticides and repellents (^a^)
38	2-Pentanol, 2-methyl-	C_6_H_14_O 102.1748	590-36-3	5.266	–	–	0.22	Bioactivity unknown
39	3-Buten-1-ol, 3-methyl or isoprenol	C_5_H_10_O 86.13	763-32-6	9.555	0.13	–	–	Terpenoid or hemiterpene alcohol compound with different physiological roles (Pandey et al., 2019)
40	Phenol	C_6_H_6_O 96.111	108-95-2	9.946	0.19	–	–	Antioxidants
41	Phenol, 2,6-dimethoxy- or syringol	C_8_H_10_O_3_ 154.1632	91-10-1	20.094	0.31	–	–	Smoky flavor nutraceutical antioxidant (Loo et al., 2008)
42	Phenol, 2,6-bis(1,1-dimethylethyl)	C_14_H_22_O 206.3239	128-39-2	29.015	0.17	–	–	Antioxidant
43	Phenol, 2,4-bis(1,1-dimethylethyl)	C_14_H_22_O 278.5	96-76-4	29.015	0.17			Allelochemical (Chen et al., 2012)
				23.947			0.38	
				23.942		1.69		
44	2-Methoxy-4- vinylphenol	C_9_H_10_O_2_ 150.1745	7786-61-0	19.126	1.24		–	Allelochemical and natural germination inhibitor (Darabi et al., 2007)
				19.126		1.26		
Alkene class of compounds								
45	1-Decene	C_10_H_20_ 140.270	872-05-9	23.460	0.68	–	–	Unknown
46	(+)-4-Carene	C_10_H_16_ 136.234	29,050-33-7	19.955	–	–	0.34	Larvicides against malaria, dengue and filariasis mosquitoes (Govindarajan et al., 2016)
47	1-Nonadecene	C_19_H_38_ 266.5050	18,435-45-5	31.567	–	–	0.96	Unknown
48	1-Pentadecene	C_15_H_30_ 210.3987	13,360-61-7	25.351	–	1.29	–	Structural element of important molecules like porphyrins, algal and insect pheromones, antitumor (^a^)
49	1-Octadecene	C_18_H_36_ 252.4784	000112-88-9	28.158	0.24	–		Antibacterial, antioxidant (Mishra and Sree, 2007) and anticancer (Lee et al., 2007)
				28.154			0.21	
50	1-Hexadecene	C_16_H_32 224.4253_	629-73-2	28.167	–	1.69	–	Unknown
51	2-Hexadecene, 3,7,11,15-tetramethyl-, [R-[R^a^,R^a^-(E)]]-	C_20_H_40_ 280.5316	014237-73-1	28.585		0.13		Antimicrobial, anticancer, anti-inflammatory and diuretic (^a^)
52	2-Tetradecene, (E)-	C_14_H_28_ 196.3721	035953-53-8	21.105	–	0.15	–	Anticancer, antimicrobial and antioxidant (Manoj et al., 2012)
53	5-Tetradecene, (E)-	C_14_H_28_ 196.3721	41,446-68-8	23.460	0.68		–	Anticancer, antimicrobial and antioxidant (Manoj et al., 2012)
				21.105	–	0.15		
Alkane class of compounds								
54	Pentadecane	C₁₅H₃₂ 212.421	629-62-9	23.676	–		0.18	Unknown
				29.503		0.70		
55	Dodecane, 1-chloro-	C_12_H_25_Cl 204.780	000112-52-7	23.101	1.17	–	–	Record not present
56	1,2-15,16-Diepoxyhexadecane	C_16_H_30_O_2_ 254.41	1,000,192-65-0	29.173	0.60	–	–	Antitumor and anti-inflammatory (^a^)
57	Octadecane	C_18_H_38_ 254.4943	593-45-3	28.239	–	0.30	–	Bioactivity not determined
58	Cyclododecane	C_12_H_24_ 168.319	294-62-2	23.291	–	0.12	–	Floral essential oil precursor to laurolactam, a precursor to the polymer Nylon-12. Antioxidant, toxic to brine shrimp larvae (Salehi et al., 2019)
59	Heneicosane	C_21_H_44_ 296.5741	629-94-7	23.359		0.61		Strong defensing activity against a number of pathogens, antifungal and antibacterial (Biswas et al., 2017)
				32.112			2.26	
60	Heptacosane	C_27_H_56_ 380.7335	593-49-7	26.898	–	–	1.25	Volatile oil but function unknown
61	Eicosane	C_20_H_42_ 282.4575	112-95-8	33.998	–	1.42		Insecticidal and antifungal (Manoj et al., 2012)
				32.839			3.49	
62	Eicosane, 2-methyl	C_21_H_44_ 296.5741	1,560-84-5	27.490	–	0.50	–	Plant insect interaction (^a^)
63	Octacosane	C_28_H_58_ 394.7601	630-02-4	27.490	–	0.40	–	Antibacterial (^a^)
				27.773			0.33	
64	1-Chloroeicosane	C_20_H_41_Cl 317	42,217-02-7	34.861	–	0.09	–	Not known
65	Pentacosane	C_25_H_52_ 352.6804	629-99-2	34.941	–	0.12		Natural wax of plants, a semiochemical, contact and volatile pheromone (Nerio et al., 2010)
				33.554			1.02	
66	Heptadecane	C_17_H_36_ 240.475	629-78-7	28.239	–	0.30	-	Essential oils
67	Nonane, 4,5-dimethyl-	C_11_H_24_ 156.3083	17,302-23-7	29.672	–	0.20	-	Not known
68	Hexadecane	C_16_H_34_ 226.4412	000544-76-3	25.460	–	0.12	-	A volatile essential oil
69	Hexadecane, 2-methyl-	C_17_H_36_ 240.4677	001560-92-5	33.038	–	–	5.08	Not found
70	Bicyclo [3.1.1] heptane, 2,6,6-trime thyl-, (1.alpha.,2.beta.,5.alpha.) or pinane	C_10_H_18_ 138.25	473-55-2	28.67	1.02		–	Highly repellant to insects (Nerio et al., 2010)
				28.687		3.27		
71	Tetratriacontane	C_34_H_70_ 478.9296	14,167-59-0	28.315	–	0.37		Antibacterial and antifungal (Mahmood et al., 2009)
				31.941			0.53	
Alkyne class of compounds								
72	1-Pentadecyne	C_15_H_28_ 208.3828	765-13-9	31.896	–	–	4.72	Compound not reported
73	9-Octadecyne	C_18_H_36_ 250.4626	35,365-59-4	31.888	3.95	–	–	Function not known
Alkylbenzene class of compounds								
74	Butylated Hydroxytoluene	C_15_H_24_O 220.3505	128-37-0	23.769	–	–	0.52	Antioxidant
75	Benzene, 1-chloro-3-methyl-	C_7_H_7_Cl 126.583	000108-41-8	10.593	0.28	–	–	Unknown
Ether class of compounds								
76	Diphenyl ether	(C_6_H_5_)_2_O 170.2072	101-84-8	21.481	–	–	0.26	Herbicide and antibacterial (Kini et al., 2010)
77	15-Crown-5	C_10_H_20_O_5_ 220.2628	033100-27-5	31.128	0.22	–	–	No records found
Heterocyclic class of compounds								
78	(-)-cis-3,4-Dimethyl-2-phenyltetra hydro-1,4-thiazine	C_12_H_17_NS 207.34	1,000,130-99-6	32.247	–	–	1.55	Antibacterial, antifungal, antiviral, analgesics, anti-inflammatory, antitumor, antitubarcular, antimalarial and anti-diabetic (Choudhary et al., 2018)
79	Pyridine-3-carboxamide, oxime, N-(2-trifluoromethylphenyl)-	C_13_H_10_F_3_N_3_O 281.23	1,594-58-7	30.548	0.98			Present in many plants but biological function not determined
				33.706		1.93		
				33.381			1.50	
80	2-Pyridinecarbohydrazonamide, N′-[(2,4-dimethoxyphenyl)methylidene]-	C_15_H_16_N_4_O_2_ 284.31		32.573	–	–	1.41	Unknown function
Carboxylic class of compounds								
81	Tetradecanoic acid or myristic acid	C_14_H_28_O_2_ 228.3709	544-63-8	32.7595	0.48			Antibiotic, larvicidal and repellent Activity (Sivakumar et al., 2011)
				27.926		0.25		
				30.404			7.90	
82	Methyl stearate	C_19_H_38_O_2_ 298.5038	112-61-8	31.710	3.47	–	–	Anti-stress role, precursor of antimicrobial and other bioactive molecules (Salehi et al., 2019)
83	Octadecanoic acid or Stearic acid	C_18_H_36_O_2_ 284.4772	57-11-4	39.95	0.54	–	–	Important long chain fatty acid, role in plant defense against pathogens (Coleman and Hudson, 2016)
84	9,12,15-Octadecatrienoic acid, (Z, Z,Z)- or linoleic acid	C_18_H_30_O_2_ 278.4296	000463-40-1	31.530	2.04	–	–	Plant defense, antimicrobial, anti- inflammatory, hypocholesterolemic and hepatoprotective
				31.520		0.88		
				31.516			1.02	
85	n-Hexadecanoic acid (palmitic acid)	C_16_H_32_O_2_ 256.424	57-10-3	30.383	4.86			Anti-inflammatory, antioxidant, Hypocholesterolemic nematicide, pesticide, antiandrogenic flavor and hemolytic (Aparna et al., 2012), 5-Alpha reductase inhibitor and potent mosquito; larvicide (Carta et al., 2017; Rahuman et al., 2000, 2009), anti-inflammatory (Vasudevan et al., 2012)
				30.442		7.72		
				30.404			7.90	
86	Dodecanoic acid or lauric Acid	C_12_ H_24_ O_2_ 200.3187	143-07-7	30.784	0.33		–	Strong bactericidal properties, inhibits uric acid production, used as urine acidifier and urine acidulant (^a^)
				25.055		0.21		
				33.778		1.02		
87	n-Dodecyl acetate or lauryl acetate	C_14_H_28_O_2_ 228.376	112-66-3	25.596	–	0.09	–	Bactericidal/fungicidal
88	4-tert-Butylcyclohexyl acetate	C_12_H_22_O_2_ 198.3019	32,210-23-4	19.570	–	–	0.13	Bioactivity unknown
89	Tetramethyl silicate	C_4_H_12_O_4_Si 152.2212	681-84-5	6.023	0.10	–	–	Relieves stress, particularly salinity stress (Sahebi et al., 2015)
90	Methyl tetradecanoate	C_15_H_30_O_2_ 242.3975	000124-10-7	27.287	–	1.14	–	Flavoring agent and fragrance

^a^ Reference source: Dr Duke's Phytochemical and Ethnobotanical Online database

### Multivariate Statistical Analyses

**FIGURE 10:**
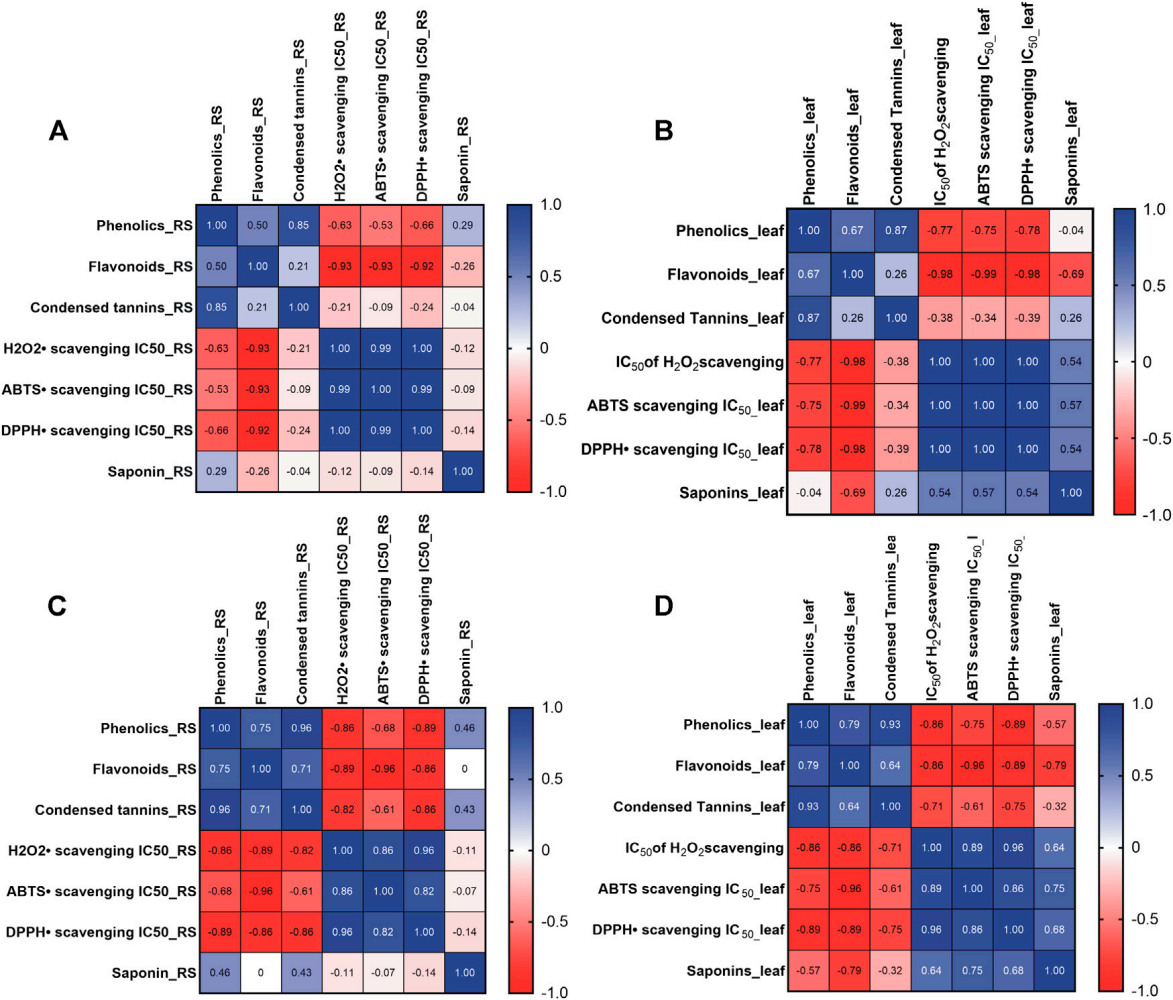
Correlation matrices showing multiple phenolics, flavonoids, condensed tannins with IC_50_ values of DPPH scavenging activities. **(A)** and **(B)** depict Pearson’s correlation matrix for RS and leaves respectively while **(C)** and **(D)** shows Spearman’s correlation for *Suaeda monoica* RS and leaves respectively. The scale on the right side shows color codes with corresponding *r* values.

To understand the correlation of the phytochemical contents (total phenolics, flavonoid, saponin and tannin), Pearson’s correlation and Spearman’s correlation were evaluated among themselves. The analysis was conducted using averaged value of each variable and the correlation matrix figures are presented in Figure 10. The IC50 values of DPPH, ABTS and H_2_O_2_ scavenging activity in different solvent extracts show a negative correlation with all the phytochemicals (phenolics, flavonoids and condensed tannins) present in respective solvent extracts indicating a positive relationship of the antioxidants with the above mentioned phytochemicals (as IC_50_ values are inversely related to antioxidant level).This study supported the previous observations that polyphenols (phenolics, flavonoids and condensed tannins) are mostly recognized for antioxidant activities. In both the analyses, except saponins, however, phenolics, flavonoids and condensed tannins maintained a positive relationship among themselves. Nevertheless, the highest level of correlation was observed between phenolics and condensed tannins with *r* = 0.85–0.87 in Pearsons matrix and *r* = 0.96–0.93 in nonparametric Spearman matrix.

**FIGURE 11:**
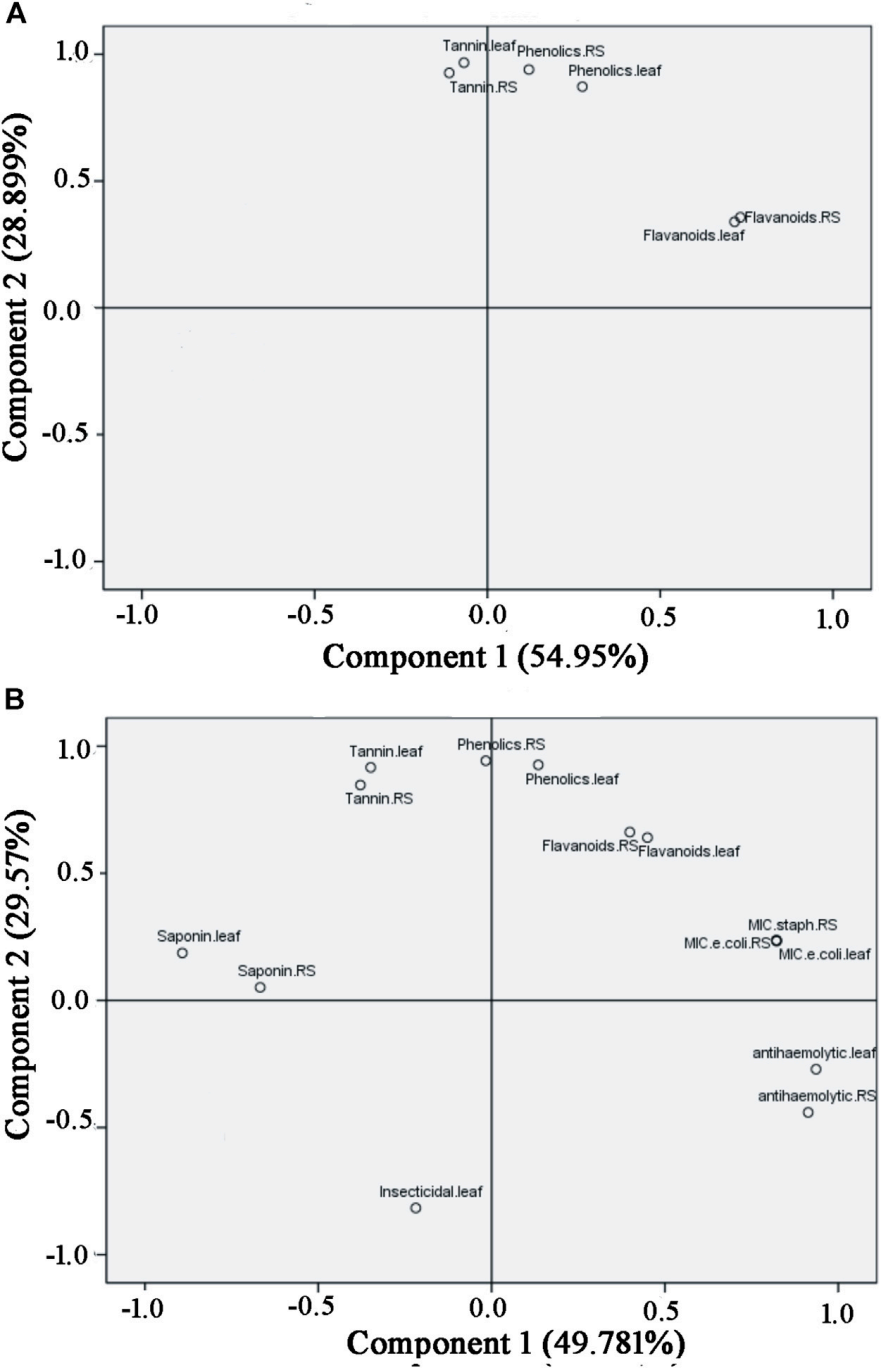
Principle component analysis showing loading plots depicting relationship of phytochemicals with the antioxidants **(A)**, relationship of phytochemicals with the bioactivities **(B)**.

Further, principal component analysis was performed with IBM SPSS software to evaluate the relationship of the phytochemicals (phenolics, flavonoids, condensed tannins and saponins) with the bioactivities like LC50 values of mosquitocidal activity, IC50 value of free radical assay, IC50 value of antihaemolytic activity and MIC values against *E. coli* and *Staphylococcus* sp*.* (Figures 11A,B). The correlation matrix of PCA shown in Figure 11A indicated that phenolics, flavonoids, condensed tannins and antioxidants are positively correlated but saponins are negatively correlated and as such confirmed the Pearson’s and Spearmen’s correlation data. Then the bioactivities data i.e., LC50 values of mosquitocidal activity, IC50 value of free radical scavenging assay, IC50 value of antihaemolytic activity and MIC values against *E. coli* and *Staphylococcus* sp*.* were analyzed (Figure 11B) to show the relationship between the bioactivities and the identified phytochemicals. It was found that insecticidal property had a negative relationship with the phytochemicals and antioxidant potential but a positive relation with the saponins. Thus, the present analysis is supporting the earlier observation regarding insect killing activities of saponins. Again, loading of MIC values displayed a positive relationship with the phenolics, flavanoids and antioxidants but a negative correlation with the condensed tannins, saponins and mosquitocidal property.

## Discussions

In view of the emergence of chronic diseases as the major causes of morbidity and mortality worldwide, there is an increasing need to focus on traditional herbal medicinal plants from efficient scientific perspectives. Most of the chronic diseases (heart disease, diabetes and other ailments) are the outcomes of several etiological factors manifesting the associated ailments (Pelkonen et al., 2014). However, preventing and treating these major chronic diseases by multiple drugs may lead to adverse interactions and side effects (Hopkins et al., 2006). Consequently, it is desirable to cover multiple targets at the same time with multiple active principles at a balanced and personalized manner. Treating a complex chronic disease would require covering multiple targets, and herbal medicines, which are chemically, complex mixtures containing multiple major and minor constituents, are often able to cover multiple potential targets. Accumulating evidences suggest that application of the multiomic methods, including genomics, epigenomics, transcriptomics, proteomics, metabolomics etc., will be of immense help in revisiting traditional medicines to get new insights and will also offer opportunities for the development of new types of medicines. It is quite important to mention that Indian subcontinent is renowned for its cultural and plant biodiversity where large number of people is still depending on traditional medicinal plants for various diseases. So, before such undisclosed ethno-pharmacological information gets lost with the loss of biodiversity, it is utmost necessary to document this conventional unique information related to the ethnic population (Ragupathy and Newmaster, 2009) and perform organized research to reestablish and validate the therapeutic potential of various medicinal plants that are not documented in the literature systematically. Nevertheless, an increasing research on medicinal plants has highlighted the importance of halophytes as one of the vast sources of polyphenols and other secondary metabolites that plants acquire in adaptive responses to stress conditions (Ksouri et al., 2012). To address the same comprehensively, the present study describes a number of investigations establishing various ethnomedicinal properties of the mangrove associate plant *Suaeda monoica* of Indian Sundarbans. The antibacterial, antifungal, allelopathic, antioxidant, mosquitocidal, antihaemolytic and antidiuretic activities rationalized its broad therapeutic potentials and thus, should be considered as one of the distinctive medicinal plants for its huge promise as a source of medicinal hub. Antioxidant ability of plant extract as determined by chemicals assays was further substantiated by biological assays, namely, DNA nick test, catalase, superoxide dismutase and ascorbate peroxidase tests indicating strong stress relieving ability of *S. monoica.* Again, like other halophytes, *S. monoica* also possesses a good amount of polyphenols, comparable with other halophytic salt marsh species (Patra et al., 2011). However, a differential distribution of these classes of compounds in polar and nonpolar solvent extracts based on their polarity (Table 4) explains the manifestation of highest antioxidant potential of the plant in the polar solvent extracts that has got the active presence of higher amount of polyphenols. Further, based on the reported literature on antimicrobial compounds (Cowan, 1999), in the present study, the phytochemicals related to antimicrobial activity were distributed in the polar ester and acid groups of compounds as well as in nonpolar alkene and alkane groups of compounds. In this context, *n*-hexane extract was shown to comprise of significant amount of 9,17-octadecadienal (4.26%), 9,12-Octadecadienoic acid (Z,Z)-, methyl ester (1.55%), 10,13-Octadecadienoic acid, methyl ester (1.55%), heneicosane (2.26%), *cis*-3,4-dimethyl-2-phenyltetrahydro-1,4-thiazine (1.55%), octacosane (0.33%), and tetratriacontane (0.87%) which are reported as potent antimicrobial compounds (Duke, 1992; Rajeswari et al., 2013; Biswas et al., 2017; Choudhary et al., 2018). In an analogous context, other identified phytochemicals such as acetic acid (28.14%), phenol (0.19%), methylcarbamate (0.55%), phytol (1.52%), methyl stearate (3.47%), linoleic acid (2.04%) and methenamine (0.44%), reported of having antimicrobial property are present in polar methanol extracts. On the other hand, in case of intermediate polar ethyl acetate extract, the presence of phytol (1.13%), 5-Tetradecene, (E)-(0.15%), pentadecane (0.70%), dodecanoic acid or lauric acid (0.21%), palmitic acid (7.72%), linoleic acid (0.88%), tetradecanoic acid or myristic acid (0.25%) etc have been revealed as possible antimicrobial compounds. So, at this point, we cannot pinpoint a particular compound or a group of phytochemicals responsible for the highest antimicrobial property present in the *n*-hexane extract as observed by the MIC assay. GC–MS identified few phenolics compounds including phenol (0.19%), 2,6-dimethoxyphenol or syringol (0.31%), 2,4-bis(1,1-dimethylethyl)phenol (1.69%), 2,6-bis(1,1-dimethylethyl)phenol (0.17%), 2,4-bis(1,1-dimethylethyl)phenol (0.17%) and 2-methoxy-4-vinylphenol (1.26%) which share reports as potent antioxidants (Foti, 2007; Loo et al., 2008; Jeong and Jeong, 2010; Zhao et al., 2020). In addition, many other molecules present in Table 4, share reports of acting as antioxidant molecules. The different types of bioactivities including strong antihaemolytic activity must be due to rich polyphenolics content and antioxidant molecules of the plant.

While dealing with plant-derived phenolics, it should be kept in mind that over 80,000 different types of phenolics with wide range of chemical structures have been found in plant kingdom (Dai and Mumper, 2010). This plant must have a large number of phenolic compounds but only a very small fraction was identified by GC–MS. Possibly, due to limitations of the analysis of non-derivatized polar organics by GC–MS, relatively polar plant phenolics such as the derivatives of benzoic acid (gallic acid), and the derivatives of cinnamic acid (coumaric acid, caffeic acid and ferulic acids) that were detected by HPLC remained undetected in GC–MS. It may be mentioned here that gallic acid and catechin have antioxidant and anticancer properties (Dai and Mumper, 2010) while caffeic acid was reported to reduce the acute immune and inflammatory response (Huang et al., 1988). Besides, rutin has antioxidant, antihypertensive, antiviral and antiplatelet properties (Ganeshpurkara and Salujaa 2017) while ferulic acid is a strong antioxidant (Srinivasan et al., 2007). Apart from significant antioxidant potential (Gramza et al., 2005), gallic acid has also been shown to possess antimicrobial activity against human pathogens (*Staphylococcus aureus*, *Corynobacterium accolans, Candida albicans*) as well as plant pathogen (*Erwinia carotovora*) (Fogliani et al., 2005). Other studies have reported cytotoxic activity of gallic acid (Kaur et al., 2005). Again, since gallic acid can act as a nucleophile, it can therefore scavenge electrophilic mutagens (Hour et al., 1999). Molecules detected by HPLC like gallic acid (Badhani et al., 2015), ferulic acid (Srinivasan et al., 2007), and rutin were reported to possess strong antioxidant activity (Al-Rejaie et al., 2013).

In addition to phenolics, saponins are another group of important phytochemicals, reported to possess antibacterial, antifungal and/or anti-insecticidal property (Wina et al., 2005; Golawska et al., 2006). Saponins are amphiphilic glycosides, where the polar moieties that include sugars (pentoses, hexoses or uronic acids), are connected covalently to a nonpolar group that includes an aglycone, known as sapogenin, which can have either a streroid, alkaloid or triterpene backbone depending on the nature of the aglycone (Almutairi and Alia, 2014). The antimicrobial activity of saponins and its presence in various solvent extracts may be accounted for highest antimicrobial activity in *n*-hexane extract of *S. monoica.*

Previous studies had reported insecticidal and mosquito larvicidal activities of inland halophytes (Thangam and Kathiresan, 1991) including some mangroves of Indian Sundarbans (Suresh et al., 2017; Balakrishnan et al., 2016). Apart from the observed insecticidal and mosquitocidal activities in solvent extract of *S. monoica,* presence of many phytochemicals (4-Carene, eicosane, eicosane, 2-methyl, methyl ester of carbamic acid, and pinane) with reports of insecticidal and mosquitocidal properties have been registered (Table 4). So, *S. monoica* can be included in the growing list of plants with significant mosquito larvicidal activity for the production of insect and mosquito repellents. In addition to the antimicrobial and insecticidal properties, nearly monotypic species distribution of the plant made us curious to know about allelochemic property of the plant, which has not been reported so far. Allelopathic compounds of plants are secondary compounds that exert stimulatory, neutral, or inhibitory activity to the receiving target plant or plant organ. As seeds are an important plant organ and most sensitive to allelochemicals, seed germination is the most widely used bioassay for allelopathic studies. As expected, a significant dose-dependent phytotoxic activity of the polar extracts of the plant was observed toward tomato seeds (as representative glycophyte) and Amaranthus (as widely known weed), establishing the herbicidal and weedicidal potential of the mangrove associate. Our finding of the allelopathic potential of the plant was supported by the detection of various phytochemicals like furfural, 2-furanmethanol, 2,4-di-tert-butylphenol, 2-methoxy-4-vinylphenol, diphenyl ether etc (Table 4), reported to have herbicidal activity. A high amount of saponin is related to the haemolytic property i.e., lysis of normal and red blood cells (Gauthier et al., 2009), but antihaemolysis assay detected the significant presence of anti-haemolytic property in the polar solvent extract of the plant, which was again supported by the identification of the relevant anti-inflammatory compounds by GC–MS (Table 4). Apart from above, phytol and 7,9-di-tert-butyl-1-oxaspiro (4,5)deca-6,9-diene-2,8-dione, 3,7,11,15-tetramethyl-2-hexadecene found in this study, had also been reported as diuretic agents (Duke, 1992). In addition, GC–MS data also showed the presence of probable hepatoprotective molecules as well as antitumor and anticancer or cancer preventive biomolecules (Table 4). From the above comprehensive biochemical, functional and chemometric studies, it became clear that the plant *S. monoica* is a wonderful phytomedicinal plant and has got a rich reservoir of structurally diverse molecules of therapeutic importance.

## Conclusion

The traditional practices of using *S. monoica* to treat several ailments have been explored systematically by performing a number of activity screening experiments with the solvent extracts of the leaf and root-shoot tissues of the plant. In the present study, a broad range of medicinal property was exhibited by this phytomedicinal plant and a polarity-based distribution of plant components in various solvents revealed a differential level of bioactivities. Further the bioactivities were supported by chemometric identification of a number of individual and classes of compounds that were reported as phytochemicals from various other plants and identified as bioactive molecules. Thus, a combination of antibacterial, antifungal, allelopathic, antioxidant, mosquitocidal, antihaemolytic and antidiuretic potentials present in a single plant *S. monoica* is quite significant, providing scientific justifications to the folkloric uses of this halophytic plant. Finally, being a rich source of many bioactive compounds, this lesser known ethnomedicinal plant should be explored further for potential application in the pharmaceutical and nutraceutical fields.

## Data Availability

The raw data supporting the conclusions of this article will be made available by the authors, without undue reservation, to any qualified researcher.
