# Supplementary material for: Evaluation of Phytochemicals and Bioactive Properties in Mangrove Associate Suaeda monoica Forssk. ex J.F.Gmel. of Indian Sundarbans
Source: Front Pharmacol. 2021 Mar 10;12:584019. doi: 10.3389/fphar.2021.584019 (PMC8006309; doi:10.3389/fphar.2021.584019)
Supplement: Supplementary file 1 [file datasheet1.pdf]

Supplementary material

Evaluation of Phytochemicals and Bioactive Properties in Mangrove Associate *Suaeda monoica* Forssk. ex J.F.Gmel. of Indian Sundarbans

*Madhumita Roy and Tapan K. Dutta**

Department of Microbiology, Bose Institute, Kolkata, West Bengal, India

Tables

Table S1 Qualitative phytochemical analysis of different solvent extracts of leaf and root-shoot samples of *S. monoica.*

Solvent	Phenolics		Flavonoids		Alkaloids		Terpenoid		Tannin		Saponin		Cardiac glycoside		Reducing sugar		Pholbatannin		Steroid	
	leaf	RS	leaf	RS	leaf	RS	leaf	RS	leaf	RS	leaf	RS	leaf	RS	leaf	RS	leaf	RS	leaf	RS
Aqueous	++	++	++	++	++	++	+	+	+	+	++	++	++	++	++	++	+	+	-	-
Methanol	++	++	++	++	++	++	++	+	++	++	+	+	+	+	+	+	+	+	-	-
DMSO	++	++	++	++	++	++	+	+	+	+	+	+	++	++	+	+	+	+	+	+
Ethyl Acetate	++	++	++	++	+	+	+	+	-	-	+	+	+	+	+	+	+	+	++	++
Hexane	+	+	-	-	+	-	-	-	-	-	+	+	-	-	+	-	+	+	++	+

According to the intensity of the color reaction, the results have been indicated as very high (+++); high (++); low (+) and not detected (-); DMSO, dimethylsulfoxide

Table S2 Mosquito larvicidal activity of the leaf of *S. monoica.*

**Extraction solvent**	**LC_{50} (LCL-UCL) mg/l**	**LC_{90} (LCL-UCL) mg/l**	**Effectiveness**
Methanol	49.329 (44.64-56.82)	57.046 (56.25-58.69)	effective
Aqueous	68.17 (66.66-75.0)	132.92 (122.28-148.02)	effective
DMSO	87.21 (83.33-96.15)	192.85 (168.75-202.5)	effective
Hexane	520.83 (446.428-625.00)	1071.42 (1022.72-1125)	ineffective
Acetone	98.69 (83.33-113.63)	195.65 (187.50-204.54)	effective
Ethyl Acetate	175.80 (144.23-203.33)	237.31 (230.11-253.125)	less effective
Chloroform	330.90 (312.50-375.0)	675.09 656.25-684.78	ineffective

LCL, lower confidence limit (95%); UCL, upper confidence limit (95%). Degree of freedom was four in all. The p-value is <0.00001. The result was found significant at $p < 0.05$.

Figures

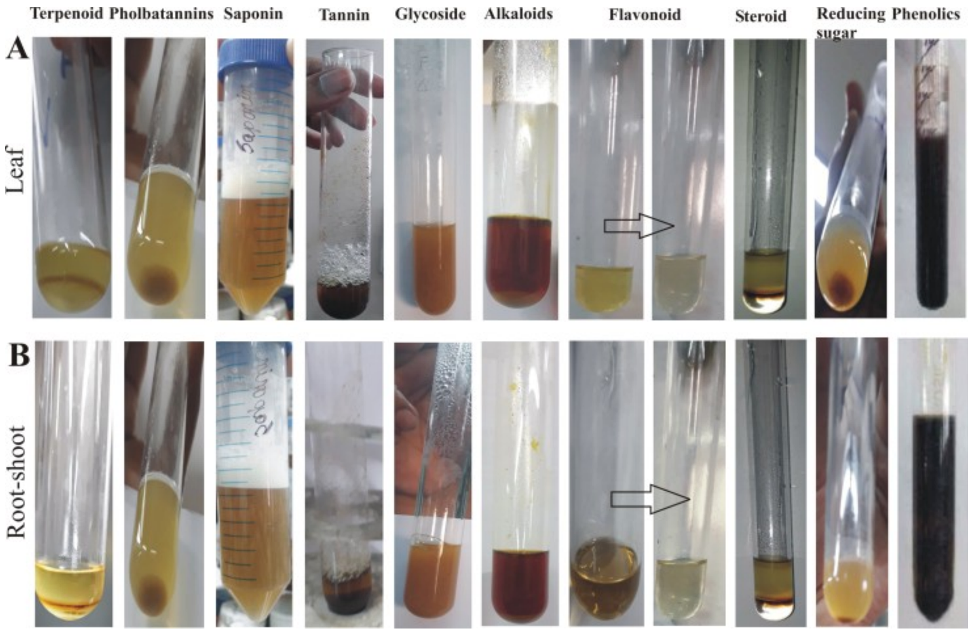

Figure S1 Assay mixtures detecting the presence of phytochemicals in methanol extract of *S. monoica*. **(A)** Leaf. **(B)** Root-shoot.

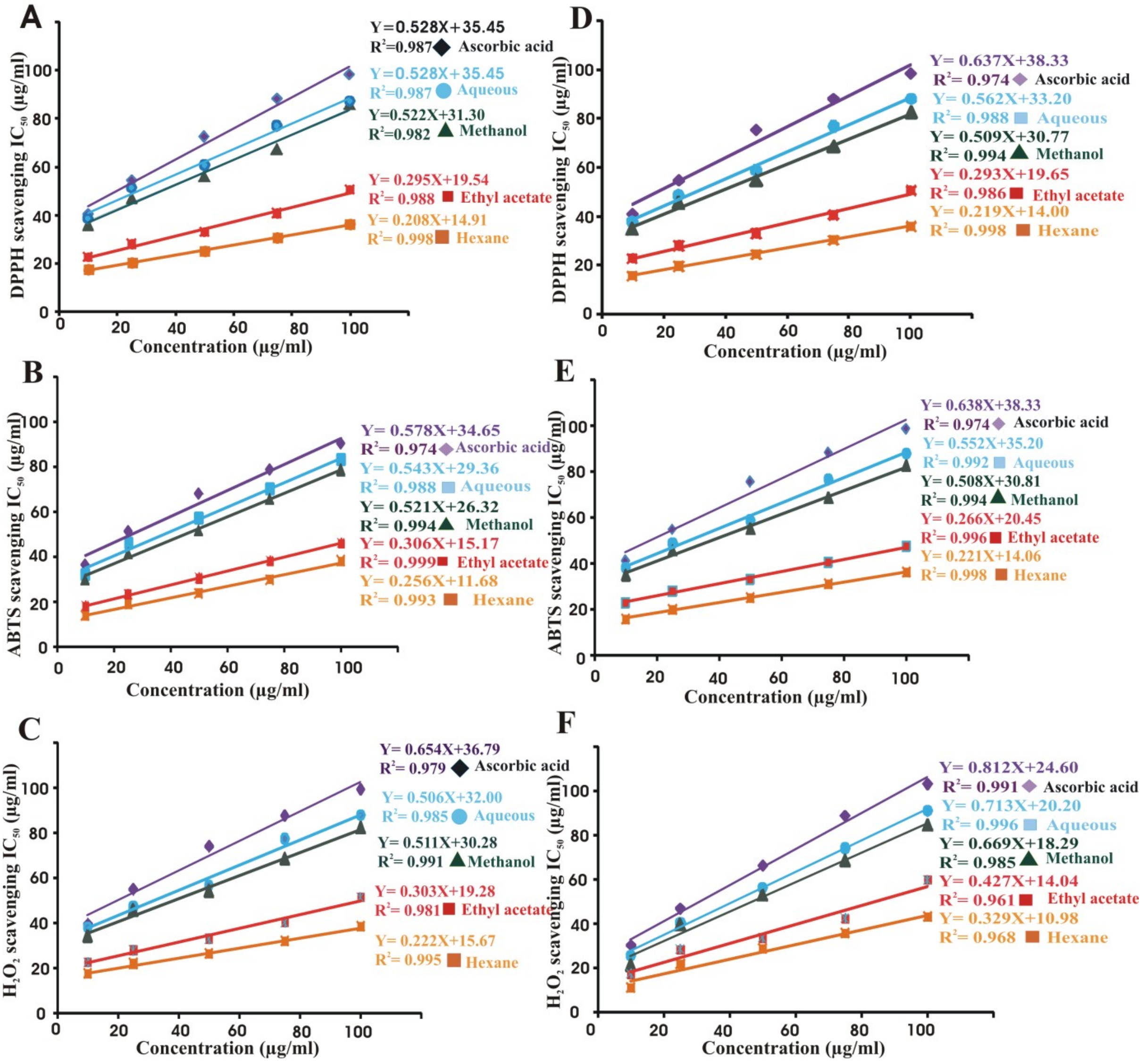

Figure S2 Antioxidant activities of root-shoot extracts (A-C) and leaves (D-F). The figures show the linear regression graphs of DPPH radical scavenging (A and D), ABTS radical scavenging (B and E) and H_2O_2 radical scavenging (C and F). The figures were constructed taking mean values of percent inhibitions obtained with different concentrations of the extracts against the free radicals.

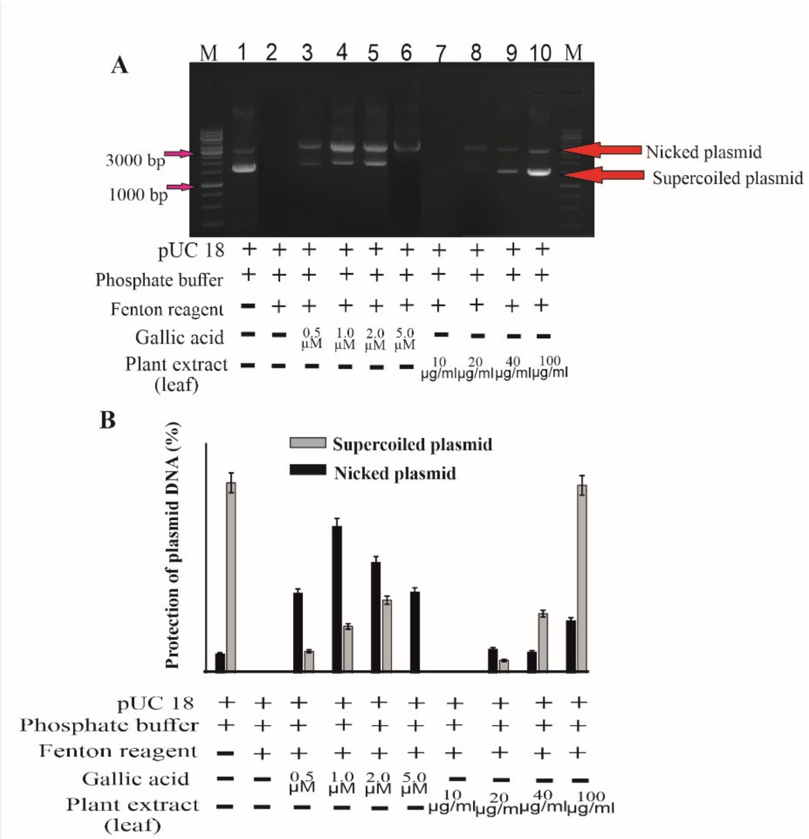

C

	CAT	APX	SOD
	Unit/mg Protein	Unit/mg Protein	Unit/mg Protein
**Root**	44.27c.	5.54c	44.09a
**Shoot**	30.13a	3.02a	52.62b
**Leaf**	35.56b	3.95b	66.65c

Figure S3(A) Agarose gel image of pUC 18 plasmid before and after exposure to Fenton reagents and protective actions of standard antioxidants and plant extracts; Lane 1, pUC plasmid DNA; Lane 2, pUC 18 DNA + Fenton reagents, negative control; Lanes 3-6, pUC plasmid DNA + Fenton reagent + 0.5-5 µM gallic acid, positive control; Lanes 7–10, pUC plasmid DNA + Fenton reagent + 10-100 µg/ml concentrations of plant leaf extracts.(B) Percentage quantification of supercoiled and nicked plasmid forms shown as bar diagram based on agarose gel image. Data represent the mean (±S.E.) of six individual replicates. (C) The activities (U mg^{-1} protein) of superoxide dismutase (SOD), ascorbate peroxidise (APX) and catalase (CAT) (n=6) present in root, shoot and leaf. Different letters presented in activity columns indicate significance levels based on t-test at $p < 0.05$.

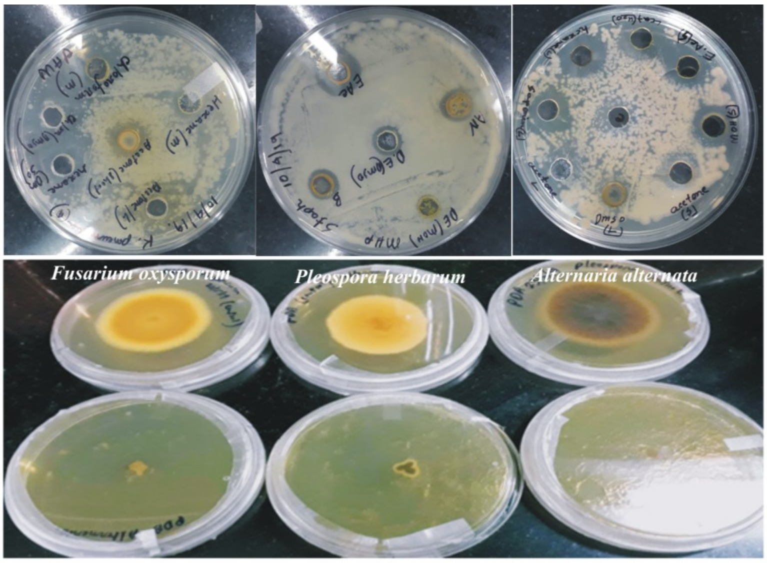

Figure S4 Antimicrobial activity of *S. monoica*. Plates in the first row show the zone of inhibitions in agar well-based antibacterial assay against three pathogens (*Escherichia coli, Staphylococcus aureus and Klebsiella pneumonia*). The second and third rows show the antifungal plug assay against three plant pathogens (*Fusarium oxysporum, Plospora herbarum* and *Alternaria solani*) where same amount of mycelial plug was put in the test (third row) and control plates (second row). After three days of incubation, mycelial spreading was observed in almost one half of the diameter of the control plates containing methanol while no growth was detected in the test plates containing 500 mg/ml plant extracts in methanol.

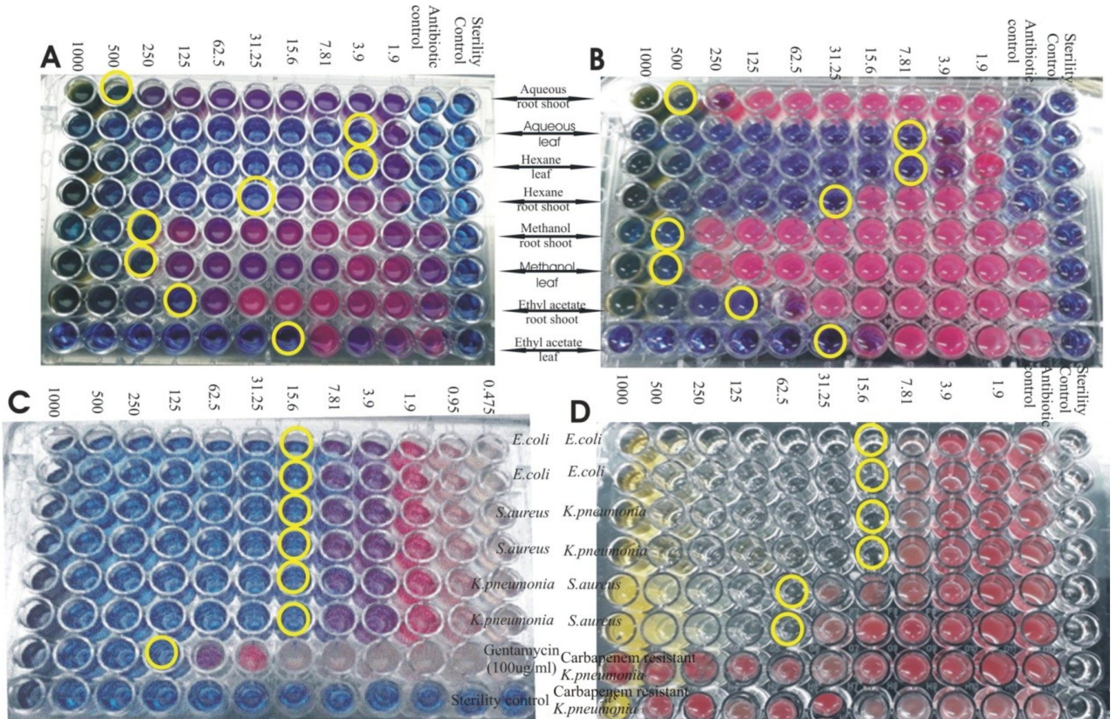

Figure S5 Resazurin microtitre assay for the determination of Minimum Inhibitory Concentration (MIC). Profiles of various extracts against *E. coli* (A) and *Staphylococcus*

aureus (B) are depicted along with chloroform extract against different pathogens (C) and extracted saponin against different pathogens (D). Pink color indicates high growth while blue means inhibition of growth and violet indicates little growth marking transition from growth to no growth. Wells, marked with circles indicated corresponding MIC values.

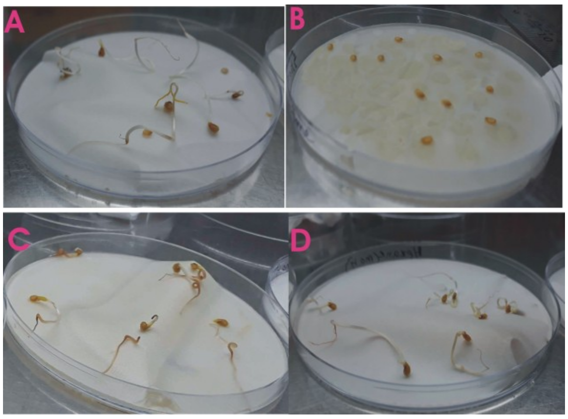

Figure S6 Bioassay of seed germination and seedling growth of Tomato. Germination status on 7th day, in presence of autoclaved water (negative control) **(A)** 500 µg/ml methanol extract **(B)** 500 µg/ml ethyl acetate extract **(C)** and 500 µg/ml *n*-hexane extract **(D)** of root-shoot sample of *S. monoica*.

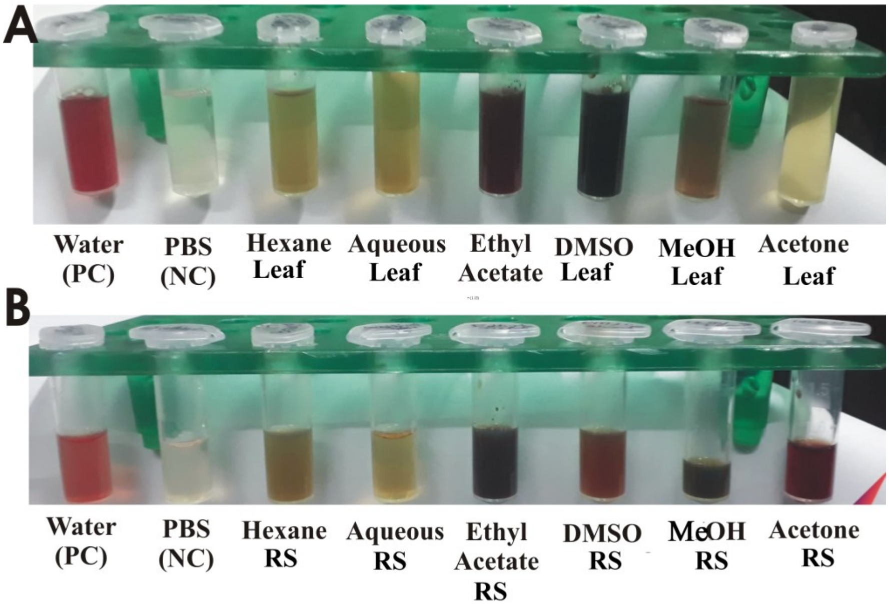

Figure S7 Depiction of anti-haemolysis profile of the supernatant after reaction of 10% RBC suspension with 1000 µg/ml concentration of leaf **(A)** and root-shoot **(B)** extracts of *S. monoica*. Positive control (water) shows 100% haemolysis while ethyl acetate, DMSO and acetone root-shoot fractions also showed considerable haemolysis. But other solvent extracts inhibited haemolysis to appreciable extents exhibiting erythrocyte membrane stabilization property. DMSO, dimethyl sulfoxide; MeOH, methanol; RS, root-shoot.

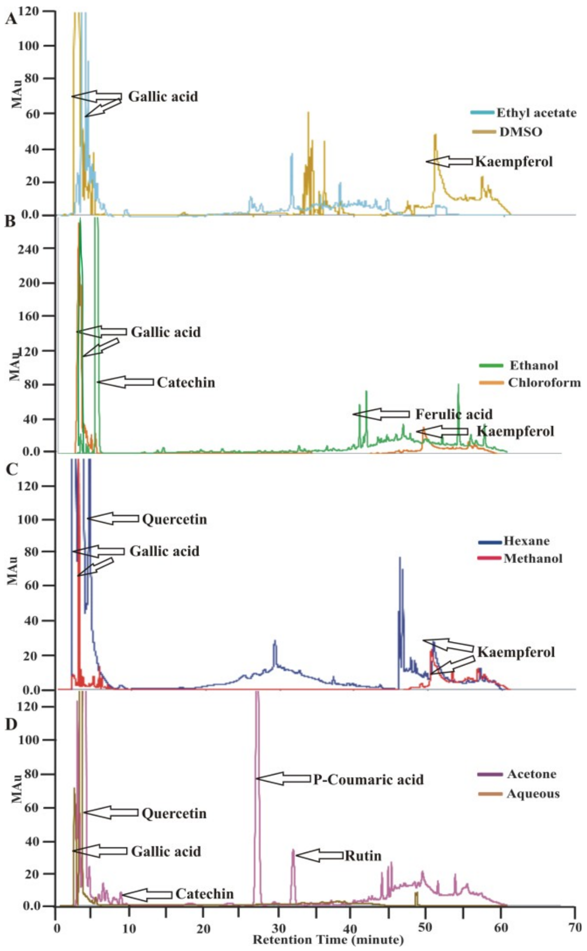

Figure S8 HPLC profiles of different solvent extracts.

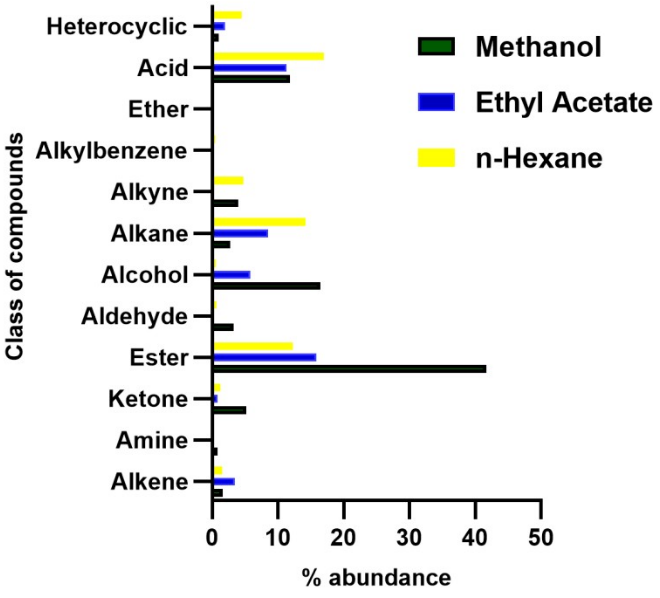

Figure S9 The bar diagram shows the % abundance of different groups of phytochemicals in various solvent extracts.

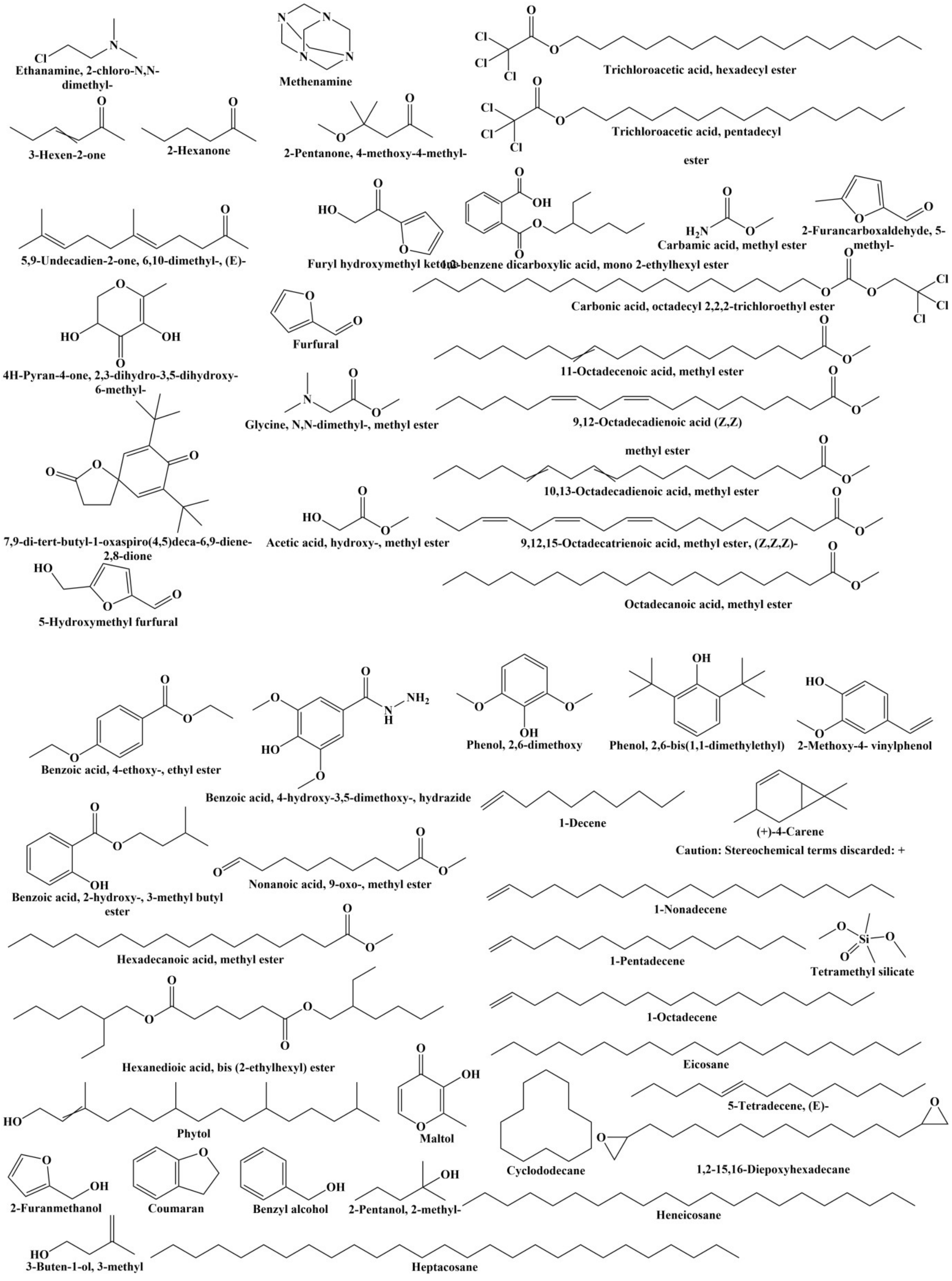
Ethanamine, 2-chloro-N,N-dimethyl-
Methenamine
Trichloroacetic acid, hexadecyl ester
3-Hexen-2-one
2-Hexanone
2-Pentanone, 4-methoxy-4-methyl-
Trichloroacetic acid, pentadecyl ester
5,9-Undecadien-2-one, 6,10-dimethyl-, (E)-
Furyl hydroxymethyl ketone
1,2-benzene dicarboxylic acid, mono 2-ethylhexyl ester
Carbamic acid, methyl ester
2-Furancarboxaldehyde, 5-methyl-
Carbonic acid, octadecyl 2,2,2-trichloroethyl ester
4H-Pyran-4-one, 2,3-dihydro-3,5-dihydroxy-6-methyl-
Furfural
11-Octadecenoic acid, methyl ester
Glycine, N,N-dimethyl-, methyl ester
9,12-Octadecadienoic acid (Z,Z) methyl ester
10,13-Octadecadienoic acid, methyl ester
7,9-di-tert-butyl-1-oxaspiro(4,5)deca-6,9-diene-2,8-dione
Acetic acid, hydroxy-, methyl ester
9,12,15-Octadecatrienoic acid, methyl ester, (Z,Z,Z)-
5-Hydroxymethyl furfural
Octadecanoic acid, methyl ester
Benzoic acid, 4-ethoxy-, ethyl ester
Benzoic acid, 4-hydroxy-3,5-dimethoxy-, hydrazide
Phenol, 2,6-dimethoxy
Phenol, 2,6-bis(1,1-dimethylethyl)
2-Methoxy-4- vinylphenol
1-Decene
(+)-4-Carene
Caution: Stereochemical terms discarded: +
Benzoic acid, 2-hydroxy-, 3-methyl butyl ester
Nonanoic acid, 9-oxo-, methyl ester
1-Nonadecene
Hexadecanoic acid, methyl ester
1-Pentadecene
Tetramethyl silicate
Hexanedioic acid, bis (2-ethylhexyl) ester
1-Octadecene
Eicosane
Phytol
Maltol
Cyclododecane
5-Tetradecene, (E)-
1,2-15,16-Diepoxyhexadecane
2-Furanmethanol
Coumaran
Benzyl alcohol
2-Pentanol, 2-methyl-
Heneicosane
3-Buten-1-ol, 3-methyl
Heptacosane

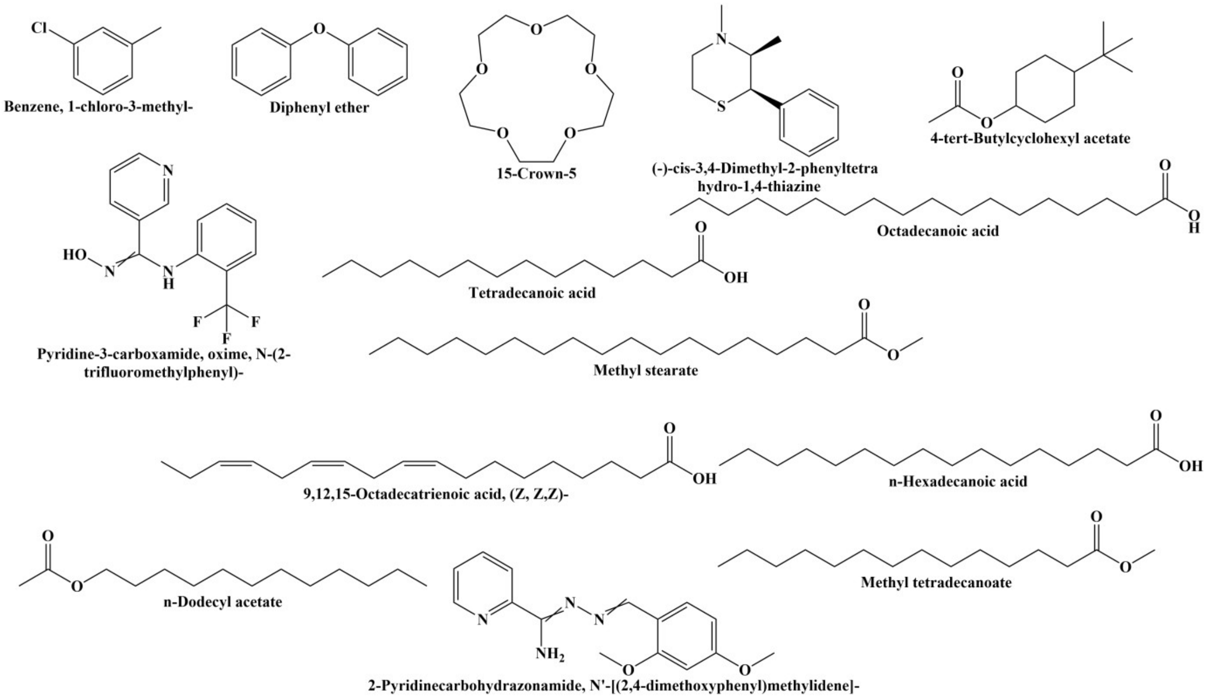

Figure S10 Chemical structures of various compounds identified by GC-MS from solvent extracts of *S. monoica*. The structures were derived based on NIST/Wiley mass spectral library search.
